# Human cytomegalovirus pUL97 upregulates SOCS3 expression via transcription factor RFX7 in neural progenitor cells

**DOI:** 10.1371/journal.ppat.1011166

**Published:** 2023-02-08

**Authors:** Xian-Zhang Wang, Le Wen, Yue-Peng Zhou, Sheng-Nan Huang, Bo Yang, Shuang Cheng, Wen-Bo Zeng, Meng-Jie Mei, Jin-Yan Sun, Xuan Jiang, Han Cheng, Min-Hua Luo

**Affiliations:** 1 State Key Laboratory of Virology, Wuhan Institute of Virology, Chinese Academy of Sciences, Wuhan, China; 2 University of Chinese Academy of Sciences, Beijing, China; 3 The Joint Center of Translational Precision Medicine, Guangzhou Institute of Pediatrics, Guangzhou Women and Children Medical Center, Guangzhou, China; 4 Wuhan Institute of Physics and Mathematics, Innovation Academy of Precision Measurement Science and Technology, Chinese Academy of Sciences, Wuhan, China; 5 Shanghai Public Health Clinical Center, Fudan University, Shanghai, China; State University of New York Upstate Medical University, UNITED STATES

## Abstract

Congenital human cytomegalovirus (HCMV) infection causes severe damage to the fetal brain, and the underlying mechanisms remain elusive. Cytokine signaling is delicately controlled in the fetal central nervous system to ensure proper development. Here we show that suppressor of cytokine signaling 3 (SOCS3), a negative feedback regulator of the IL-6 cytokine family signaling, was upregulated during HCMV infection in primary neural progenitor cells (NPCs) with a biphasic expression pattern. From viral protein screening, pUL97 emerged as the viral factor responsible for prolonged SOCS3 upregulation. Further, by proteomic analysis of the pUL97-interacting host proteins, regulatory factor X 7 (RFX7) was identified as the transcription factor responsible for the regulation. Depletion of either pUL97 or RFX7 prevented the HCMV-induced SOCS3 upregulation in NPCs. With a promoter-luciferase activity assay, we demonstrated that the pUL97 kinase activity and RFX7 were required for SOCS3 upregulation. Moreover, the RFX7 phosphorylation level was increased by either UL97-expressing or HCMV-infection in NPCs, suggesting that pUL97 induces RFX7 phosphorylation to drive SOCS3 transcription. We further revealed that elevated SOCS3 expression impaired NPC proliferation and migration *in vitro* and caused NPCs migration defects *in vivo*. Taken together, these findings uncover a novel regulatory mechanism of sustained SOCS3 expression in HCMV-infected NPCs, which perturbs IL-6 cytokine family signaling, leads to NPCs proliferation and migration defects, and consequently affects fetal brain development.

## Author summary

HCMV is the most common infectious cause of birth defects, and medical intervention options are still limited due to a poor understanding of the underlying molecular mechanisms. Fetal brain development involves complicated cytokine signaling pathways that are tightly controlled. Here, we show that HCMV infection in NPCs upregulates SOCS3, a negative feedback regulator of IL-6 cytokine family signaling. We demonstrate that the viral protein pUL97 interacts with and probably phosphorylates the host transcription factor RFX7 to drive SOCS3 transcription. Elevated SOCS3 level alters NPC cell fates and reduces NPC migration, presumably contributing to the HCMV-induced fetal brain defects. Our work reveals a new regulatory mechanism for SOCS3 expression in NPCs and provides insights into the neuropathogenesis induced by congenital HCMV infection.

## Introduction

Congenital human cytomegalovirus (HCMV) infection is the leading cause of birth defects, affecting 0.5–3% of newborns worldwide [1,2]. Up to 20% of congenital-infected infants will show symptoms or develop long-term neurologic conditions, including microcephaly, cerebral palsy, hearing loss, etc [3–6]. These neurological impairments adversely affect children’s ability to acquire language, communication, and social skills, imposing considerable economic and psychological burdens on the affected families. Currently, FDA-approved vaccines to prevent congenital HCMV infection and effective therapeutic options to reverse the neurologic sequelae are still unavailable [7–10], making it imperative to decipher the molecular mechanisms underlying the infection-induced abnormal brain development.

Congenital HCMV infection preferentially targets the neural progenitor cells (NPCs) residing in the ventricular zone (VZ) and subventricular zone (SVZ) in the fetal brain, which are pluripotent and can differentiate into neuronal and glial cells [11,12]. In the early embryonic stages, NPCs first give rise to neurons during neurogenesis and later glia during gliogenesis, delicately controlled by the coordination between cell-intrinsic regulators and extracellular signals [11,13,14]. As a critical internal regulator responsible for NPC self-renewal and differentiation, STAT3 can be activated through JAK/STAT3 pathway in response to extracellular IL-6 family cytokines, including cardiotrophin-1, neuropoietin, leukemia inhibitory factor (LIF), and IL-6. Genetically disrupting this signaling pathway by knocking out gp130 or LIFRβ, the components of the IL-6 family receptor, impairs NPC self-renewal, and deletion of STAT3 changes the expression of several NPC markers [15]. The JAK/STAT3 pathway is inhibited by several neurogenic factors during neurogenesis. However, later gliogenic signals downregulate these factors and revive the signaling. The activated JAK/STAT3 pathway consequently promotes astrogliogenesis through a positive autoregulatory loop [11,13,16].

Several negative feedback mechanisms exist to prevent toxic side effects of sustained activation of cytokine signaling. The IL-6/JAK/STAT3 signaling induces the expression of suppressor of cytokine signaling 3 (SOCS3) that consequently disrupts the pathway by simultaneously interacting with JAK and IL-6 receptor gp130 [17,18]. Because of its critical role in IL-6 cytokine signaling, SOCS3 has been intensively studied in immune cells and is considered a major regulator of infection and inflammation [19]. Growing evidence suggests that SOCS3 is also vital for NPCs maintenance and differentiation [20] and participates in the regulation of astrogliogenesis [13]. Overexpression of SOCS3 can rescue long-term self-renewal of SOX2-deleted NPCs [21]. In addition, SOCS3 contributes to the delicate regulation of NPC self-renewal and differentiation by negatively modulating the LIF signal [22]. SOCS3 induces neurite differentiation of neural stem cells (NSCs) via PI3K/AKT pathway and promotes neuronal cell survival under oxidative stress [23]. Interestingly, SOCS3 deletion can promote neural repair and induce sustained axon regeneration in the adult central nervous system [24].

Given SOCS3’s multiple roles in regulating infection and neural development, we wondered whether HCMV infection in the neural system affects SOCS3 expression and how the altered SOCS3 level contributes to the HCMV-induced brain maldevelopment. Here, we analyzed the SOCS3 expression pattern in HCMV-infected NPCs, which displayed a pattern of biphasic upregulation. Further mechanistic studies revealed that a host transcription factor, regulatory factor X 7 (RFX7), is required for viral protein pUL97-mediated upregulation of *SOCS3* transcription. As for the impacts of the SOCS3 upregulation on NPC biology, we demonstrate that elevated SOCS3 expression reduced NPC proliferation and migration *in vitro*, and SOCS3-expressing NPCs exhibit migration defects *in vivo*. This work reveals a new regulatory mechanism between viral and host factors and provides insight into neuropathogenesis caused by congenital HCMV infection.

## Results

### HCMV infection upregulates SOCS3 expression in NPCs

**Fig 1:**
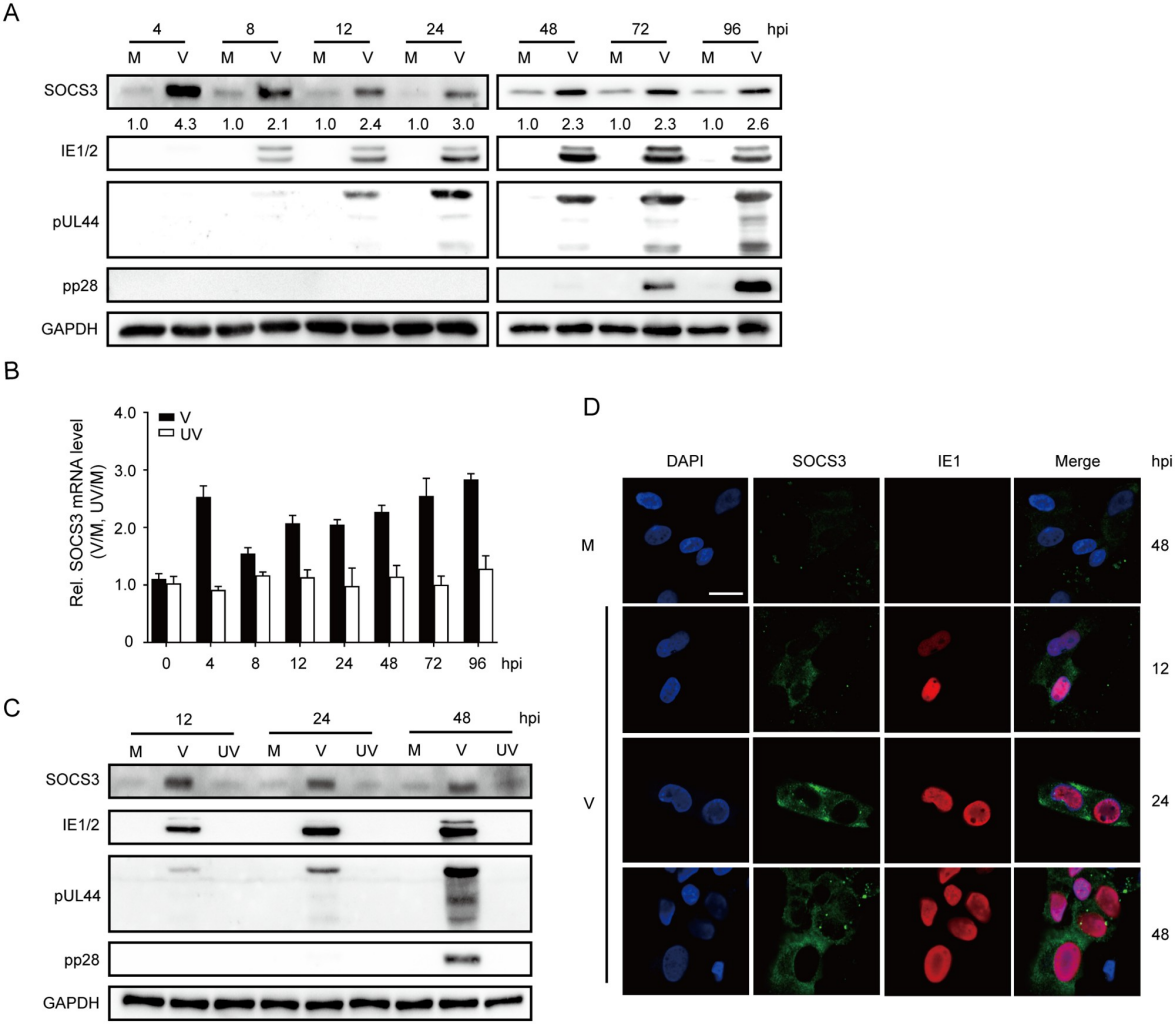
HCMV infection up-regulates SOCS3 expression in NPCs. NPC monolayers were mock-infected (M) or infected with either HCMV (V) or UV-inactivated HCMV (UV) at an MOI of 3, samples were collected at the indicated time points. **(A)** SOCS3 and viral protein levels. Viral protein levels of IE1/IE2, pUL44, and pp28 were determined by western blotting. Relative SOCS3 levels (V versus M) were listed below the blots. GAPDH served as a loading control and was used for protein quantification normalization. **(B)** SOCS3 mRNA levels. The levels of SOCS3 mRNA were determined by qRT-PCR, normalized to GAPDH, and relative SOCS3 mRNA levels (V versus M, UV versus M) were shown. The data are from three independent experiments and presented as average ± SD. **(C)** SOCS3 and viral protein levels. SOCS3, IE1/IE2, pUL44, and pp28 protein levels were determined by western blotting. GAPDH served as a loading control. **(D)** Cellular distribution of SOCS3 and IE1. NPCs grown on poly-D-lysine-coated coverslips were infected, coverslips were harvested at the indicated time points, and distribution of SOCS3 and IE1 were assessed by immunofluorescence assay with antibodies against SOCS3 (green) and IE1 (red), and the nuclei were counterstained with DAPI (blue). Scale bar, 15μm.

NPCs are fully permissive to HCMV infection [25]. To evaluate the effects of HCMV on SOCS3 expression in NPCs, we first examined SOCS3 protein levels in HCMV-infected NPCs. IE1/IE2, pUL44, and pp28 were analyzed to indicate the immediate early, early, and late viral gene expression, respectively. As shown in Fig 1A, the relative SOCS3 protein levels in HCMV- versus mock-infected NPCs increased dramatically at 4 hours post-infection (hpi) to 4.3-fold, quickly dropped to 2.1-fold at 8 hpi, and then slowly rose to 2.6-fold at 96hpi. We also confirmed this biphasic increase of SOCS3 expression at the transcription level, in which SOCS3 mRNA level quickly peaked at 4hpi, dropped at 8 hpi, and trended up again slowly till the end of the infection cycle (Fig 1B). We next asked whether viral gene expression is required for the induced SOCS3 expression. NPCs were infected by an ultraviolet (UV) -inactivated HCMV (UV-HCMV) with abrogated viral transcriptional activity. Unlike the observations in wild-type HCMV infection (V), the mRNA (Fig 1B) and protein (Fig 1C) levels of SOCS3 remained unperturbed during UV-HCMV infection, indicating newly synthesized viral products are required for SOCS3 regulation.

In addition, we inspected the impact of HCMV infection on SOCS3 cellular distribution. SOCS3, as a cytoplasmic protein, was hardly detected in mock-infected NPCs. However, SOCS3 in the cytoplasm was gradually increased by HCMV infection, and the viral IE1 protein was restricted to the nucleus as usual (Fig 1D). These data demonstrate that HCMV infection enhances SOCS3 expression without affecting its subcellular localization.

### Sustained upregulation of SOCS3 requires pUL97

**Fig 2:**
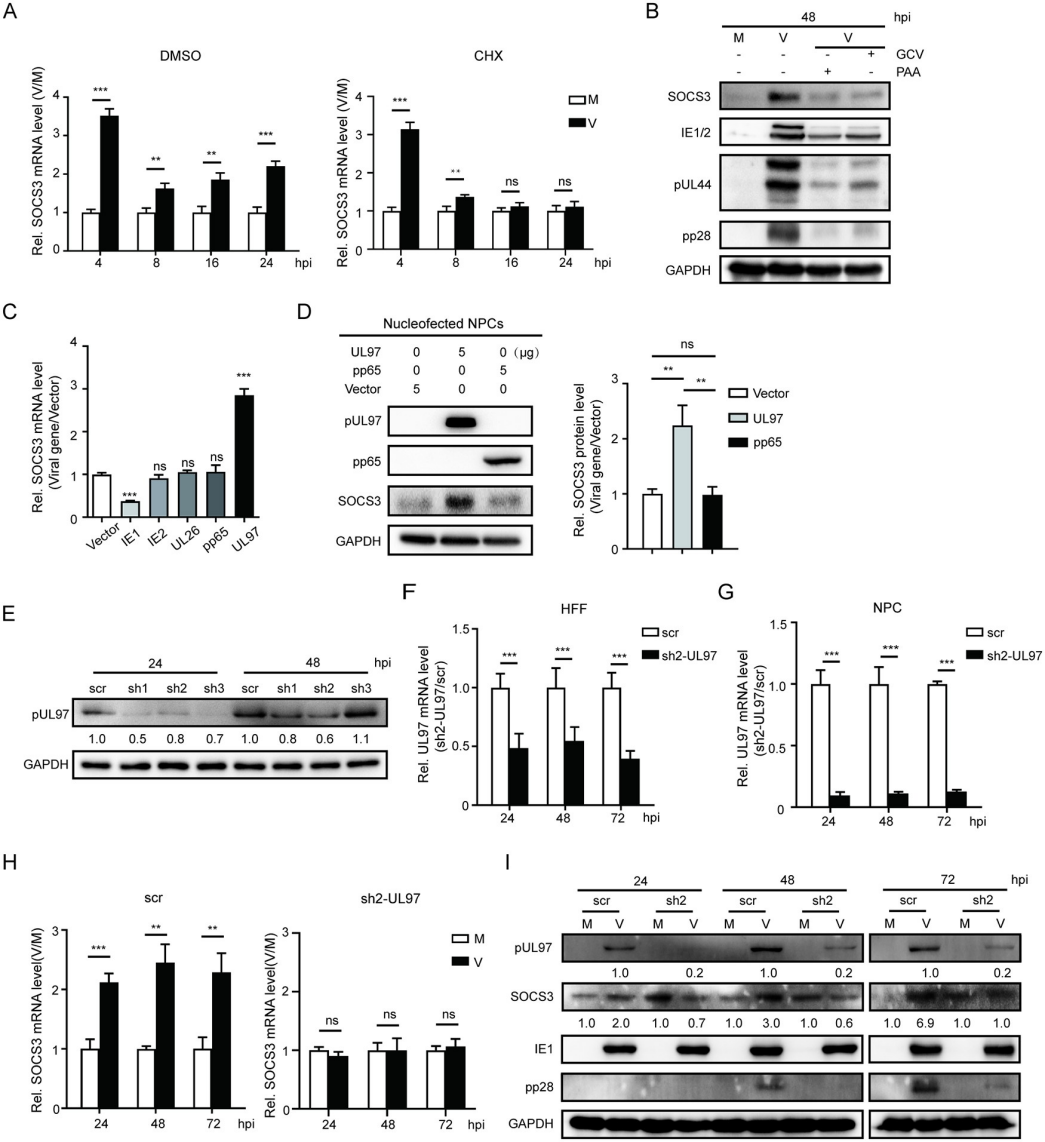
pUL97 upregulates SOCS3 expression. **(A)** Effects of protein synthesis inhibitor CHX treatment on SOCS3 mRNA levels. NPCs were treated with CHX for 1 h before infection, followed by mock- or HCMV-infection at an MOI of 3. Cells were harvested at 4, 8, 16, and 24 hpi. SOCS3 mRNA levels were determined by qRT-PCR and normalized to GAPDH. **(B)** Effects of viral replication inhibitors on SOCS3 expression. NPCs were pretreated with GCV or PAA for 1 h before infection, followed by mock- or HCMV-infection at an MOI of 3. SOCS3 and representative viral protein levels (IE1/IE2, pUL44, and pp28) at 48 hpi were determined by western blotting. GAPDH served as a loading control. **(C)** Screening of viral proteins for SOCS3 upregulation. NPCs were transduced with lentiviruses expressing candidate viral genes at an MOI of 1. The levels of SOCS3 mRNA were determined by qRT-PCR normalized to the GAPDH, and then compared to the corresponding vector control. **(D)** The effect of ectopic UL97 expression on SOCS3 protein level. NPCs were transfected with 5 μg of PHAGE-pp65, PHAGE-UL97, or PHAGE vector by nucleofection, and cells were collected at 48 h post-transfection for western blotting analysis. GAPDH served as a loading control. **(E)** Knock-down efficiency of shRNA-UL97 candidates in pUL97 levels. HFF cells were transduced with lentiviruses expressing shRNA-UL97-1 (sh1), shRNA-UL97-2 (sh2), shRNA-UL97-3 (sh3), or shRNA-scramble (scr). At 48 h post-transduction, cells were infected with HCMV at an MOI of 3 and collected at 24 or 48 hpi. pUL97 levels were determined by western blotting. pUL97 levels relative to scr controls were listed below the blots. GAPDH served as a loading control and was used for normalization. **(F)** UL97 mRNA knockdown efficiency by sh2 in HFF. HFF cells transduced with sh2 or scr-expressing lentiviruses were infected with HCMV as in (E). UL97 mRNA levels were determined by qRT-PCR at the indicated times. **(G-I)** Effects of sh2 on UL97 and SOCS3 expression in NPCs. NPCs were transduced with sh2 or scr-expressing lentiviruses and cultured for 48h. The transduced NPCs were then mock- or HCMV-infected at an MOI of 3, and collected at 24, 48, and 72 hpi for qRT-PCR and western blotting analysis. The SOCS3 and pUL97 levels were normalized to GAPDH; relative protein levels are listed below the blots (for pUL97, comparisons were made between the infected cells at each timepoint; for SOCS3, comparisons were made between each M/V pair). The data in (A, C, D, F-H) are from three independent experiments and presented as average ± SD. Significance was tested with student’s t-test; ** p<0.01, *** p<0.001, ns, no significant difference.

To determine how SOCS3 expression is regulated by HCMV infection, we first examined whether SOCS3 upregulation requires newly synthesized proteins. We treated NPCs with cycloheximide (CHX) to block *de novo* protein synthesis. The CHX treatment did not affect the first fast elevation of SOCS mRNA expression but abolished the second sustained upregulation of SOCS3 expression (Fig 2A). In addition, treating NPCs with HCMV replication inhibitors (phosphonoacetic acid [PAA] and ganciclovir [GCV]) greatly reduced the infection-induced SOCS3 protein level change compared to the control (Fig 2B). These results suggest that the first upregulation during the immediate-early phase of HCMV infection likely results from the host response to viral infection. Later, as the host response fades, newly synthesized viral proteins step in to keep SOCS3 expression at an elevated level.

Because sustained SOCS3 upregulation might affect NPC fate decisions, we focused on how the virus regulates SOCS3 gene expression in the second phase. Since SOCS3 upregulation in this phase is initiated before 12 hpi (Fig 1A) when IE and early viral genes are expressed, we evaluated several IE and early viral genes for their abilities to regulate SOCS3 expression. NPCs were transduced by lentivirus expressing the candidate viral genes, and SOCS3 mRNA levels were quantified. SOCS3 transcription was not affected by pUL83 (pp65), IE2, or pUL26. Interestingly, IE1 dramatically reduced SOCS3 mRNA to 37% of the control level, whereas pUL97 significantly increased SOCS3 mRNA level by 2.9-fold (Fig 2C). The upregulation by pUL97 was also confirmed by the SOCS3 protein level increase in the nucleofected NPCs. Ectopic UL97 expression resulted in a 2-fold change in SOCS3 protein level compared with the vector and viral protein pp65 controls, which did not affect SOCS3 expression (Fig 2D). These data demonstrate that pUL97 is sufficient to upregulate SOCS3 expression in NPCs.

To further confirm that pUL97 is the viral protein in charge of SOCS3 regulation during HCMV infection, UL97 expression was knocked down by short hairpin RNAs (shRNA). First, we designed three shRNAs (sh1-3) targeting UL97 coding regions and tested them in human foreskin fibroblasts (HFFs), using shRNA-scramble (scr) as a negative control. HFFs were transduced with shRNA-expressing lentivirus, followed by infection with HCMV at an MOI of 3. Compared with the scr control, sh2 exhibited a better silencing effect than the other two shRNAs, consistently decreasing pUL97 levels at 24 and 48 hpi (Fig 2E). The efficient UL97 knockdown by sh2 was further confirmed at the transcription level, in which around 50% decreases in mRNA level relative to the controls were detected from 24 to 72 hpi (Fig 2F). Thus, sh2 was chosen for UL97 knockdown in NPCs.

NPCs were transduced with sh2- or scr-expressing lentivirus, and UL97 knockdown was assessed. The sh2 showed a higher knockdown efficiency in NPCs than in HFFs, with ~90% reduction at the mRNA level and ~80% at the protein level (Fig 2G and 2I). These transduced NPCs were then infected with HCMV at an MOI of 3 to evaluate the role of pUL97 in SOCS3 upregulation. Consistent with the results in non-transduced NPCs, SOCS3 was upregulated during HCMV infection in scr-transduced NPCs. However, SOCS3 upregulation disappeared in NPCs with UL97 knockdown by sh2 (Fig 2H–I).

Taken together, these data demonstrate that pUL97 is the viral protein responsible for the sustained upregulation of SOCS3 during HCMV infection in NPCs.

### pUL97 kinase activity is required for SOCS3 upregulation

**Fig 3:**
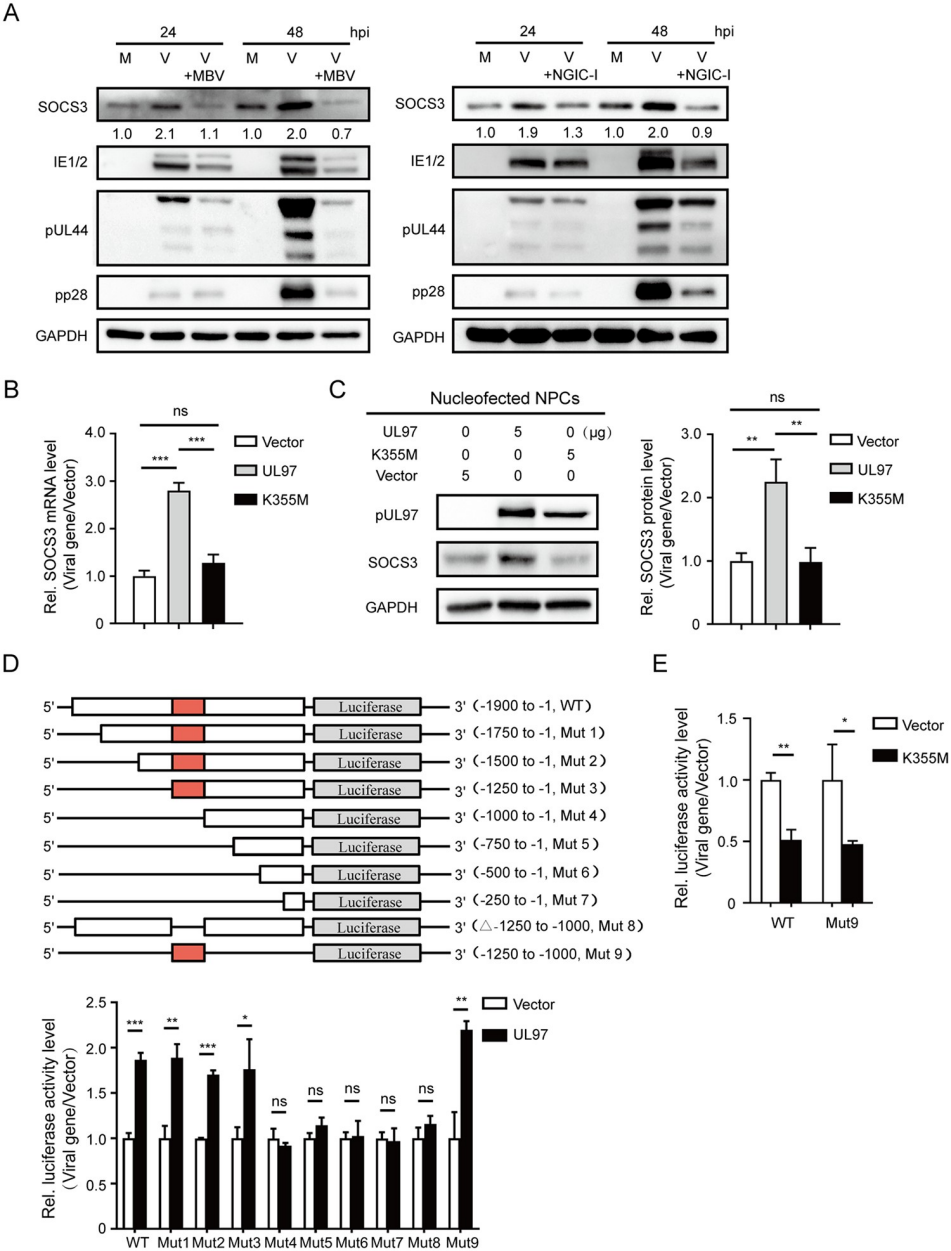
SOCS3 upregulation in NPCs requires pUL97 kinase activity. **(A)** pUL97 kinase inhibitors antagonize HCMV-induced SOCS3 upregulation. NPCs were pretreated with pUL97 kinase inhibitors (MBV, NGIC-I) or DMSO for 1 h, followed by mock- or HCMV-infection at an MOI of 3. SOCS3 and representative viral protein levels (IE1/IE2, pUL44, and pp28) at 24 and 48 hpi were determined by western blotting. SOCS3 levels relative to mock controls were listed below the blots. GAPDH served as a loading control and was used for normalization. **(B-C)** Catalytical inactive pUL97 mutant fails to upregulate SOCS3 expression. The UL97, K355M (UL97 kinase mutant) or vector was transiently expressed in NPCs via nucleofection. Samples were collected at 48h post-transfection for protein and mRNA quantification. SOCS3 mRNA levels were determined by qRT-PCR, and the protein levels were determined by western blotting. GAPDH was used for normalization. **(D)** Schematic diagram of wild-type *SOCS3* promoter and *SOCS3* promoter mutants. The UL97 or vector was cotransfected with different mutants of *SOCS3* promoter-luciferase in 293T. The luciferase activity was tested at 36h post-transfection. **(E)** Effect of K355M on *SOCS3* promoter-luciferase activity. The K355M or vector was cotransfected with wild-type *SOCS3* promoter-luciferase or Mut9 in 293T. The luciferase activity was tested at 36h post-transfection. The data in (**B-E**) are from three independent experiments and presented as average ± SD. Significance was tested with student’s t-test; * p<0.05, ** p<0.01, *** p<0.001, ns, no significant difference.

pUL97 is a viral kinase expressed in the early stage of viral infection, regulating viral nuclear egress and viral particle assembly via phosphorylation of viral and cellular substrates [26]. We next asked whether pUL97 kinase activity is required for SOCS3 regulation. We first examined the effects of two pUL97 kinase inhibitors, maribavir (MBV) and NGIC-I [26–29], on the HCMV-induced SOCS3 upregulation. NPCs were treated with MBV or NGIC-I during HCMV infection (MOI = 3). The infection significantly increased SOCS3 expression in DMSO-treated NPCs, whereas treatment of MBV or NGIC-I prevented SOCS3 upregulation, indicating pUL97 kinase activity is required for the regulation (Fig 3A). However, we noticed that the expression of other viral proteins, including IE1/2, pUL44, and pp28, was also reduced by these two inhibitors (Fig 3A). Since pUL97 is important for HCMV replication, inhibiting pUL97 function also interferes with the expression of other viral proteins, complicating the interpretation of the above result.

We, therefore, evaluated the effect of catalytically inactive pUL97 on SOCS3 expression. As the lysine residue at position 355 is essential for pUL97 kinase activity, it was mutated to methionine to generate the catalytically inactive mutant (K355M) [27,30]. Wild-type UL97 or the K355M mutant was expressed in NPCs to assess the role of pUL97 kinase activity in SOCS3 regulation. Compared with the vector control, pUL97 increased SOCS3 mRNA and protein levels to a similar extent of upregulation as observed in HCMV-infected NPCs. However, this regulation was not detected by K355M-expression (Fig 3B–3C). These data further help to confirm that the kinase activity of pUL97 is critical for the upregulation of SOCS3 in NPCs.

### pUL97 upregulates SOCS3 transcription through its promoter

To elucidate how pUL97 regulates SOCS3 transcription, we tested whether pUL97 increases *SOCS3* promoter activity. A luciferase reporter containing the *SOCS3* promoter region (-1900 to -1 bp from the transcription start sites, WT) was constructed (Fig 3D). The reporter was co-transfected with a UL97-expressing or control vector in 293T cells, and *SOCS3* promoter activity was quantified by Luciferase Assay System (Promega) at 36 h post-transfection (hpt). Similar to previous findings, the *SOCS3* promoter-driven luciferase activity was 90% higher in UL97-expressing cells than in control cells. To identify the core promoter region for pUL97-mediated regulation, a series of promoter mutants (Muts 1–9) were constructed and tested (Fig 3D, upper panel). Compared with the WT promoter, the luciferase activity of any mutant containing the segment -1250 to -1000bp (Muts 1–3 and Mut 9) was enhanced by pUL97 to the same extent. In contrast, all the mutants lacking this segment (Muts 4–8) failed to show pUL97-mediated upregulation, indicating that pUL97 utilizes the region -1250 to -1000bp to regulate SOCS3 transcription (Fig 3D, lower panel).

Given that the kinase activity of pUL97 is essential for the SOCS3 regulation, we tested whether loss of this activity would render pUL97 unfunctional in enhancing the *SOCS3* promoter activity. As shown in Fig 3E, the K355M mutant failed to increase the activities of the WT and Mut9 of *SOCS3* promoter, providing additional support to the finding that pUL97 promotes SOCS3 expression through its promoter, and the kinase activity of pUL97 is essential.

### pUL97 recruits RFX7 for SOCS3 upregulation

**Fig 4:**
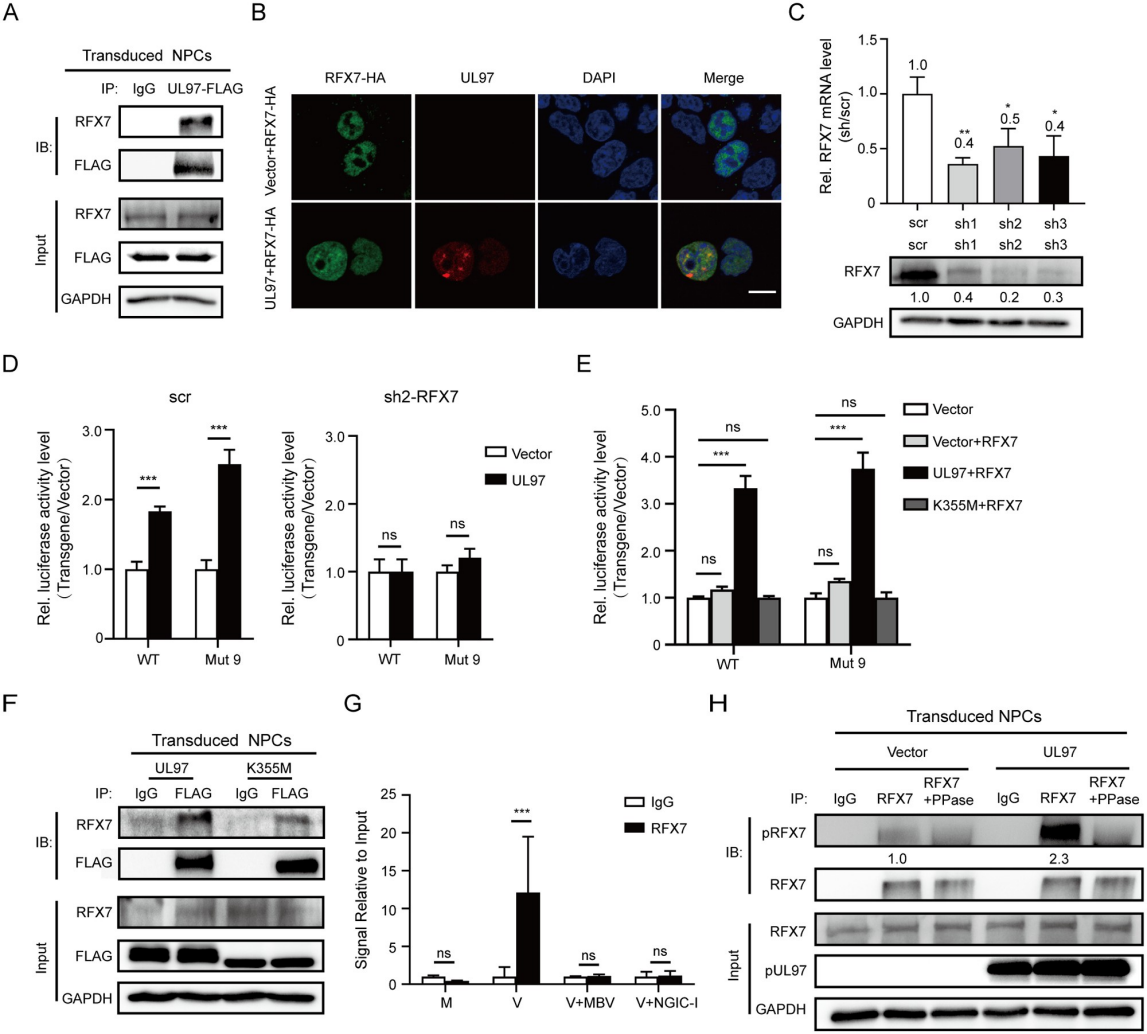
RFX7 interacts with pUL97 and is key to the pUL97-mediated *SOCS3* promoter activity enhancement. **(A)** pUL97-RFX7 interaction in NPCs. NPCs were transduced with lentivirus expressing UL97-FLAG and harvested at 72 h post-transduction for immunoprecipitation with anti-FLAG antibody. Normal IgG was used as a nonspecific antibody control. **(B)** Colocalization of pUL97 with RFX7 in nucleus. The UL97 or vector and RFX7-HA were cotransfected into 293T cells. The distributions of pUL97 and RFX7 at 48h post-transfection were assessed by immunofluorescence assay with antibodies against HA (green) and pUL97 (red). The nuclei were counterstained with DAPI (blue). Scale bar, 10μm. **(C)** Knockdown efficiencies of shRNA-RFX7 candidates. The RFX7 plasmid (1.5μg) was cotransfected with 1.5μg plasmid expressing shRNA-RFX7-1 (sh1), shRNA-RFX7-2 (sh2), shRNA-RFX7-3 (sh3), or shRNA-scramble (scr) in 293T, and cells were collected at 48h post-transfection. RFX7 mRNA levels were determined by qRT-PCR. RFX7 protein levels were determined by western blotting. RFX7 levels relative to scr control were listed below the blots. GAPDH served as a loading control and was used for normalization. **(D)** Effect of RFX7 knockdown by sh2 on the pUL97-mediated *SOCS3* promoter activity. WT or mut9 *SOCS3* promoter-luciferase was cotransfected with the UL97 or vector and sh2 or scr in 293T. The luciferase activity was tested at 36 h post-transfection. **(E)** Effect of RFX7 overexpression on the pUL97-mediated *SOCS3* promoter activity. RFX7 was cotransfected with WT or mut9 *SOCS3* promoter-luciferase and UL97, K355M or vector in 293T. The luciferase activity was tested at 36 h post-transfection. **(F)** K355M-RFX7 interaction in NPCs. NPCs were transduced with lentivirus expressing UL97-FLAG or K355M-FLAG and harvested at 72 h post-transduction for immunoprecipitation with an anti-FLAG antibody. **(G)** NPCs were pretreated with pUL97 kinase inhibitor (MBV or NGIC-I) or DMSO for 1 h, followed by mock- or HCMV-infection at an MOI of 3 for 48 h. Chromatin was then immunoprecipitated with RFX7 antibody or IgG antibody for qPCR assay using primers targeting the -1254 to -1178 region of *SOCS3* promoter. **(H)** pUL97 increases RFX7 phosphorylation in NPCs. NPCs were transduced with lentivirus expressing UL97 or the control, and cells were harvested at 72h post-transduction. RFX7 was immunoprecipitated with the anti-RFX7 antibody, followed by treatment with or without PPase. Then the phospho-Ser/Thr antibody was used to visualize phosphorylated RFX7. Normal IgG was used as a nonspecific antibody control. RFX7, pRFX7, and pUL97 were determined by western blotting; GAPDH served as a loading control. The ratios of pRFX7 to RFX7 in the IP samples were calculated and normalized to the value in the control group, and the numbers are listed below the blots. Data in (**C-E and G**) are from three independent experiments and presented as average ± SD. Significance was tested with student’s t-test for (**C and D**) and ANOVA for (**E and G**); * p<0.05, ** p<0.01, *** p<0.001, ns, no significant difference.

To our best knowledge, there has been no report of direct interaction between pUL97 and DNA so far. Thus, we speculated that it likely recruits a host transcription factor for the regulation. To identify the potential transcription factor, we enriched host proteins that interacted with ectopically-expressed pUL97-FLAG in NPCs by immunoprecipitation assay (IP) with an anti-FLAG antibody, and subjected them to liquid chromatography-tandem mass spectrometry (LC-MS/MS) analysis. RFX7 emerged as the top transcription factor candidate (S1 Table), and its interaction with pUL97 was confirmed by IP in NPCs (Fig 4A) and by colocalization analysis in 293T cells (Fig 4B).

To explore RFX7’s role in SOCS3 upregulation by pUL97, RFX7 expression was silenced by RNA interference. First, three shRNAs (sh1 to 3) targeting different RFX7 coding regions were evaluated in 293T cells. Because sh2 exhibited decent silencing effects at both mRNA and protein levels (Fig 4C), it was chosen for further assays. With RFX7 knockdown in 293T cells, ectopic expression of UL97 became incapable of increasing the luciferase expression driven by the *SOCS3* promoter WT or Mut 9 (Fig 4D), indicating that RFX7 is critical for *SOCS3* promoter-driven gene expression. To test whether RFX7 can increase the promoter activity, the WT or Mut 9 reporter was co-transfected with RFX7-expressing plasmid in 293T. RFX7 overexpression alone failed to enhance the expression of both reporters (Fig 4E). Interestingly, though the RFX7-interacting ability remains in the K355M mutant (Fig 4F), co-expressing RFX7 could not rescue the loss-of-function phenotype of the K355M mutant in the promoter activity assay. Taken together, these data strongly support that both RFX7 and pUL97 kinase activity are required for SOCS3 upregulation.

We further utilized Chromatin Immunoprecipitation (ChIP)-quantitative PCR (qPCR) assay to assess whether and where RFX7 binds to the *SOCS3* promoter. ChIP was performed using anti–RFX7 rabbit polyclonal antibody on sheared chromatin from mock- or HCMV-infected NPCs. The purified DNA was analyzed by qPCR with 13 pairs of optimized primers (S2 Table) targeting different regions of the SOCS3 promoter (-1900 to -1). Only the -1254 to -1178 region showed > 10-fold enrichment of anti-RFX7 antibody in the HCMV-infected condition. When the infected cells were treated with pUL97 inhibitor maribavir or NGIC-I, the binding of RFX7 to the -1254 to -1178 region was no longer detectable (Fig 4G). These data, together with the luciferase promoter activity assay, strongly support the direct involvement of RFX7 in pUL97-mediated SOCS3 upregulation.

We next tested whether pUL97 could phosphorylate RFX7 with its serine/threonine (ser/thr) protein kinase activity. So far, the phosphorylation sites of RFX7 remain elusive, and the phosphorylation site-specific antibodies have not been reported. Thus, an antibody recognizing a range of motifs containing phospho-Ser/Thr was used as a surrogate to assess RFX7 phosphorylation [31,32]. NPCs were transduced with lentivirus expressing UL97 or the control virus. Endogenous RFX7 was immunoprecipitated by an anti-RFX7 antibody, and then the phosphorylated RFX7 (pRFX7) was visualized by western blotting with the phospho-Ser/Thr antibody. As shown in Fig 4H, ectopic UL97 expression in NPCs had little effect on the total RFX7 protein level but boosted the pRFX7 level compared with the control. In addition, pRFX7 levels could be reduced with lambda phosphatase (PPase) treatment of the immunoprecipitated RFX7, confirming the ability of the phospho-Ser/Thr antibody to probe the phosphorylation status of RFX7.

**Fig 5:**
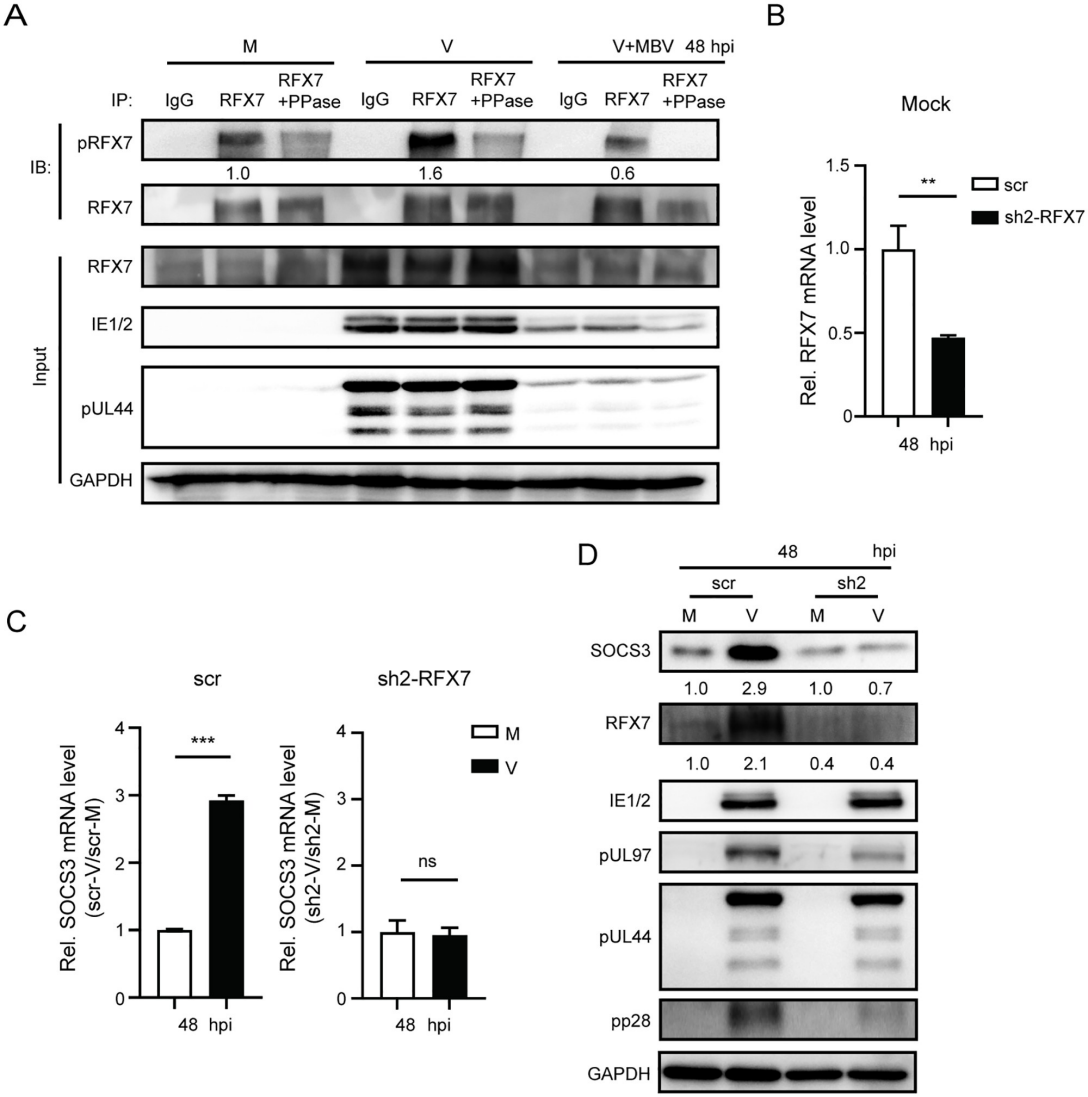
RFX7 is critical for HCMV-induced SOCS3 upregulation in NPCs. **(A)** RFX7 and its phosphorylation levels in HCMV-infected NPCs. NPCs were pretreated with MBV or DMSO for 1h, followed by mock- or HCMV-infection at an MOI of 3 for 48h. RFX7 was enriched by immunoprecipitation with the anti-RFX7 antibody. The amount of the antibody for immunoprecipitation was optimized so that the antibodies were saturated by the RFX7 proteins in the mock-infected NPCs. Thus, similar amounts of RFX7 were immunoprecipitated from different conditions. The immunoprecipitated RFX7 was treated with or without PPase. Then the phosphorylated RFX7 was visualized with the antibody targeting phospho-Ser/Thr. IE1/IE2, pUL44, and RFX7 protein levels were determined by western blotting. GAPDH served as a loading control. The ratios of pRFX7 to RFX7 in the IP samples were calculated and normalized to the value in the control group, and the numbers are listed below the blots. **(B-D)** Effects of RFX7 sh2 on RFX7 and SOCS3 expression in NPCs. NPCs were transduced with sh2 or scr-expressing lentiviruses, and then mock—or HCMV -infected at an MOI of 3. Cells were collected at 48 hpi for qRT-PCR and western blotting analysis. The SOCS3 and RFX7 mRNA levels were determined by qRT-PCR and normalized to GAPDH. The mRNA data from three independent experiments are presented as average ± SD and were analyzed by student’s t-test; ** p<0.01, *** p<0.001, ns, no significant difference. RFX7 levels relative to the scr-treated mock control and SOCS3 levels relative to the mock controls were listed below the blots.

We also examined whether HCMV infection raises the pRFX7 level in NPCs. As HCMV infection greatly increased the total RFX7 protein level (Fig 5A, input), the immunoprecipitated RFX7 amounts were adjusted to the same level between the mock and the HCMV groups for pRFX7 blotting (Fig 5A, IP). Compared with the mock-infected cells, HCMV infection noticeably increased RFX7 phosphorylation (indicated by the pRFX7/RFX7 ratio from IB) in NPCs (Fig 5A, IP). Treating the infected NPCs with pUL97 inhibitor MBV decreased RFX7 phosphorylation, suggesting that pUL97 is likely involved in this process.

To confirm the participation of RFX7 in SOCS3 upregulation during HCMV infection, RFX7 was depleted with shRNA. NPCs were transduced with RFX7 sh2 or the scr control for 48 h and then infected with HCMV at an MOI of 3. In the scr control group, HCMV infection greatly boosted RFX7 and SOCS3 expression in NPCs compared with the mock-infected cells. However, sh2 reduced RFX7 expression in NPCs (Fig 5B and 5D) and disrupted the HCMV-induced SOCS3 upregulation (Fig 5C–5D), confirming a key role of RFX7 in this regulation.

### Elevated SOCS3 expression reduces NPC proliferation, self-renewal, and migration *in vitro*

**Fig 6:**
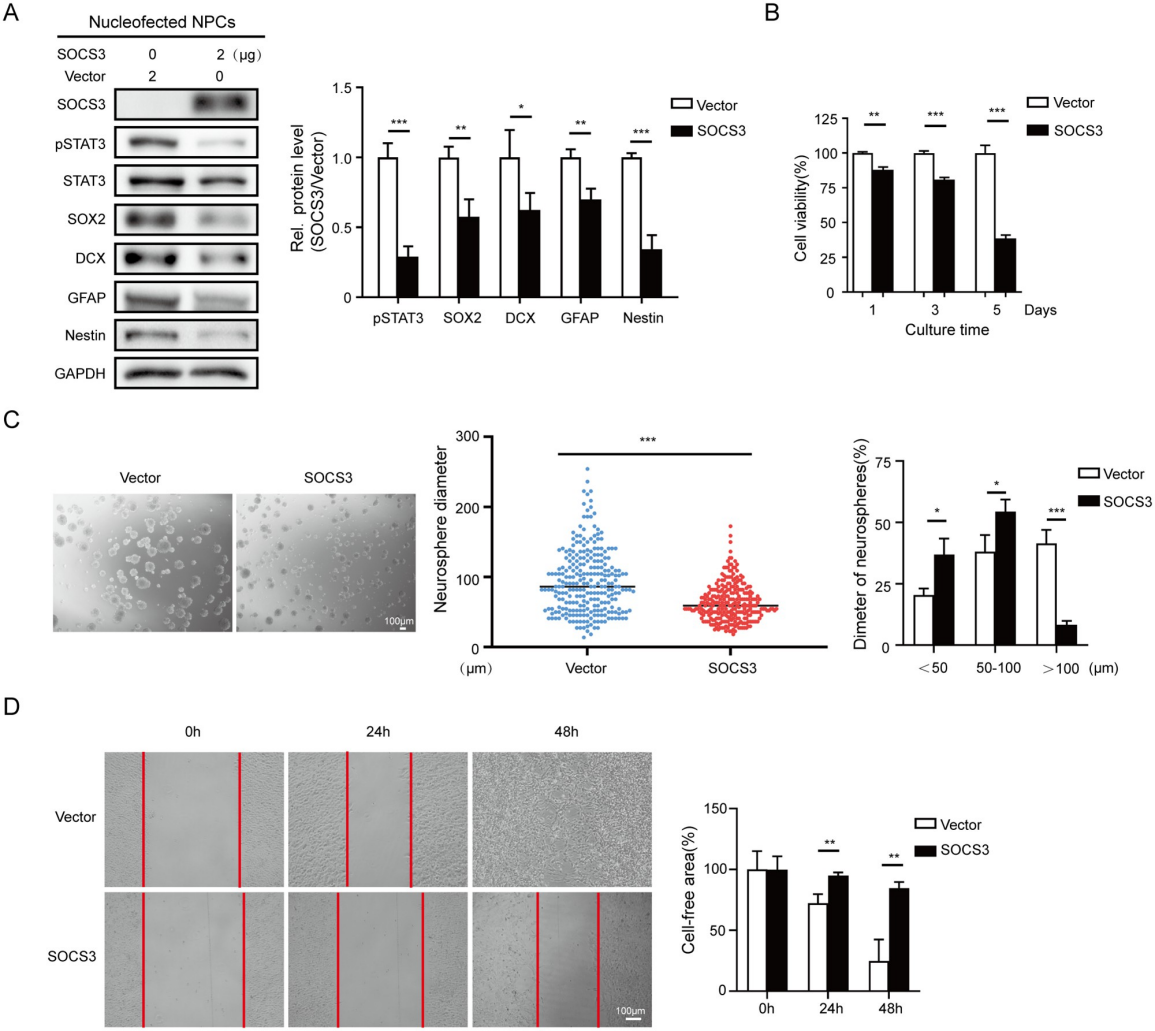
Overexpression of SOCS3 alters NPC properties. **(A)** Effect of SOCS3 on the expression of key NPC markers. NPCs nucleofected with SOCS3 or vector were cultured for 48 h and then harvested for the indicated proteins examination by western blotting. GAPDH served as a loading control. Protein levels were quantified. **(B)** Effect of SOCS3 on cell viability. NPCs were transduced with SOCS3-expressing or the control (vector) lentivirus for 48h, and then reseeded in poly-D-lysine-coated 96-well plates. NPC proliferation was assessed at 1, 3, and 5 days by WST-1 assay. **(C)** Effect of SOCS3 on neurosphere formation and growth. At 48h post-transduction, the NPCs were reseeded in uncoated 6-well plates, and images were obtained after culture for 48 h. All neurospheres were counted and categorized into three size groups (small, < 50μm; medium, 50–100μm; and large, > 100μm) from 3 random fields. Scale bars, 100μm. **(D)** Effect of SOCS3 on NPC migration. At 48h post-transduction, NPCs were reseeded in poly-D-lysine-coated 24-well plates for further culture. When cells reached confluency, a cell-free zone was created with pipette tips, and the floating and dead cells were cleared by GM wash. The same fields were imaged every 24 h and analyzed by the densitometry program (Image J). The scratch areas at the indicated times were normalized to the measured area at 0 h. Scale bar, 100μm. Data in (**A-B**) are from three independent experiments, and data in (**C**–**D**) are from three random fields or sights. All data are presented as average ± SD. Significance was tested with student’s t-test; * p<0.05, ** p<0.01, *** p<0.001.

To assess the contribution of HCMV-induced SOCS3 upregulation to the neuropathogenesis caused by congenital HCMV infection, we first examined the impact of high SOCS3 expression on the biology of NPCs. We overexpressed SOCS3 in NPCs and examined several NPC cell fate indicators. NPC markers (SOX2, GFAP, Nestin, and DCX) were analyzed by western blotting. SOCS3 overexpression alone could reduce the expression of all these markers compared with the control group (Fig 6A), which is interestingly similar to HCMV infection [33].

To inspect the effects of SOCS3 overexpression on NPC proliferation, NPCs were transduced with a SOCS3-expressing lentivirus or vector control and cultured as monolayers in poly-D-lysine-coated 96-well plates (5×10^4^ cells/well) for the WST-1 cell proliferation assay. At 1-, 3-, or 5-day post-seeding, SOCS3-expressing NPCs exhibited gradual reduction in cell growth to 88.0%, 80.9%, and 38.6% of the control levels, respectively (Fig 6B). Alternatively, transduced NPCs were cultured in uncoated 6-well plates (1×10^6^ cells/well) for the neurosphere formation and growth assessment, in which SOCS3-expressing NPCs formed smaller neurospheres with reduced diameters than the control cells (Fig 6C). The neurospheres were further categorized into three groups by diameter (small, < 50μm; medium, 50–100μm; large, >100μm). Compared with the control, substantially fewer SOCS3-expressing NPCs formed large neurospheres (41.5% versus 8.3%) (Fig 6C), indicating that elevated SOCS3 expression impairs NPC self-renewal.

Besides, we evaluated the impact of SOCS3 overexpression on NPC migration with the scratch assay. The transduced NPCs were cultured in poly-D-lysine-coated 24-well plates (1×10^6^ cells/well). After the cells formed a confluent monolayer, cell-free zones were created with pipette tips. The dynamics of NPC migration toward the cell-free region were monitored every 24 h using time-lapse microscopy, and the scratch areas were analyzed by a densitometry program (Image J). In the vector control group, the cell-free zone gradually shrank to 72.3% at 24 h and 24.8% at 48 h relative to the area at 0 h. In contrast, the migration of the SOCS3-expressing NPCs was dramatically slower, and the region remained largely cell-free at 24 h and 48 h, which were 95.0% and 84.7% of the area at 0 h, respectively (Fig 6D).

These results demonstrate that increased SOCS3 decreases the proliferation, neurosphere formation, and migration capabilities of NPCs.

### SOCS3-exprssing NPCs exhibit migration defect during fetal brain development *in vivo*

**Fig 7:**
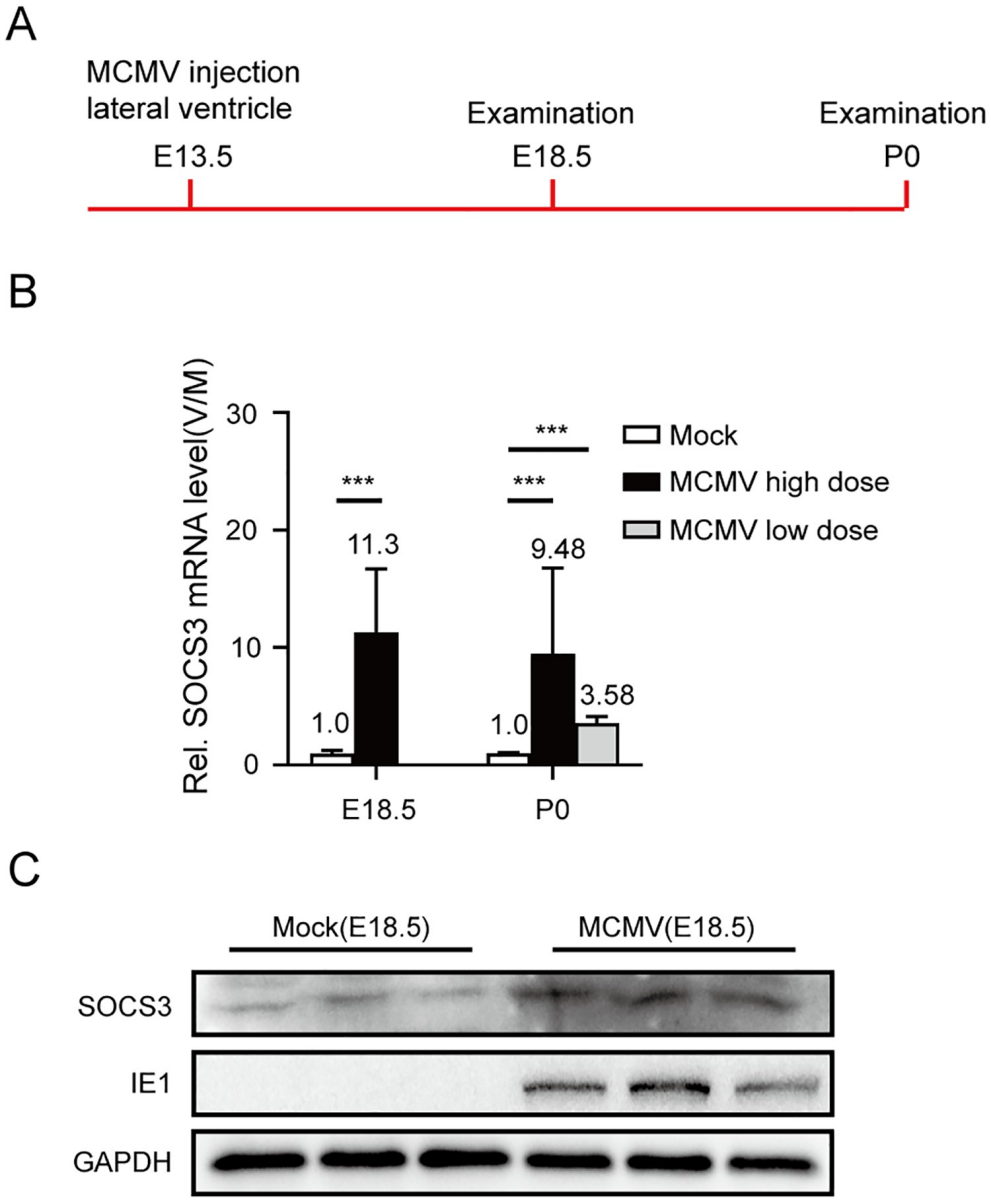
MCMV infection upregulates SOCS3 expression in mouse fetal brains. **(A)** Timeline of congenital MCMV infection and examination. Lateral ventricle injections of MCMV (high dose: 1×10^5^ pfu; low dose: 40 pfu) or mock were carried out at embryonic day 13.5 (E13.5). Brain samples of E18.5 fetuses *via* cesarean section and newborns at day 0 postnatal (P0) were collected for mRNA or protein analyses. **(B)** SOCS3 mRNA levels. SOCS3 mRNA levels were quantified by qRT-PCR and normalized to GAPDH. The data from three independent experiments (at least three fetuses/experiment/group) were analyzed by student’s t-test, and results are presented as average ± SD. *** p<0.001. **(C)** SOCS3 and viral protein levels. SOCS3 and IE1 protein levels from the E18.5 brains infected with high dosage MCMV were determined by western blotting. GAPDH served as a loading control.

To study the role of SOCS3 in neuropathology caused by congenital CMV infection, we first sought to confirm whether CMV infection upregulates SOCS3 expression *in vivo*. Mouse fetuses were infected with murine CMV (MCMV) *in utero* by direct injection of the virions into the lateral ventricle at embryo day 13.5 (E13.5) and harvested by cesarean section at E18.5 or postnatal day 0 (P0) for qPCR and western blotting analysis (Fig 7A). At E18.5, SOCS3 mRNA level was 11.3-fold higher in the high dose (1×10^5^ pfu/fetus) MCMV-infected brains than in the mock-infected brains (Fig 7B), and the upregulation was also confirmed at the protein level (Fig 7C). Even with a very low dose (40 pfu) infection, we observed a 3.6-fold increase of SOCS3 transcription in the infected brains compared with the control at P0, while the high dose led to a much bigger change of 9.5-fold (Fig 7B). Together, these results confirm that congenital MCMV infection induces significant SOCS3 upregulation *in vivo*.

**Fig 8:**
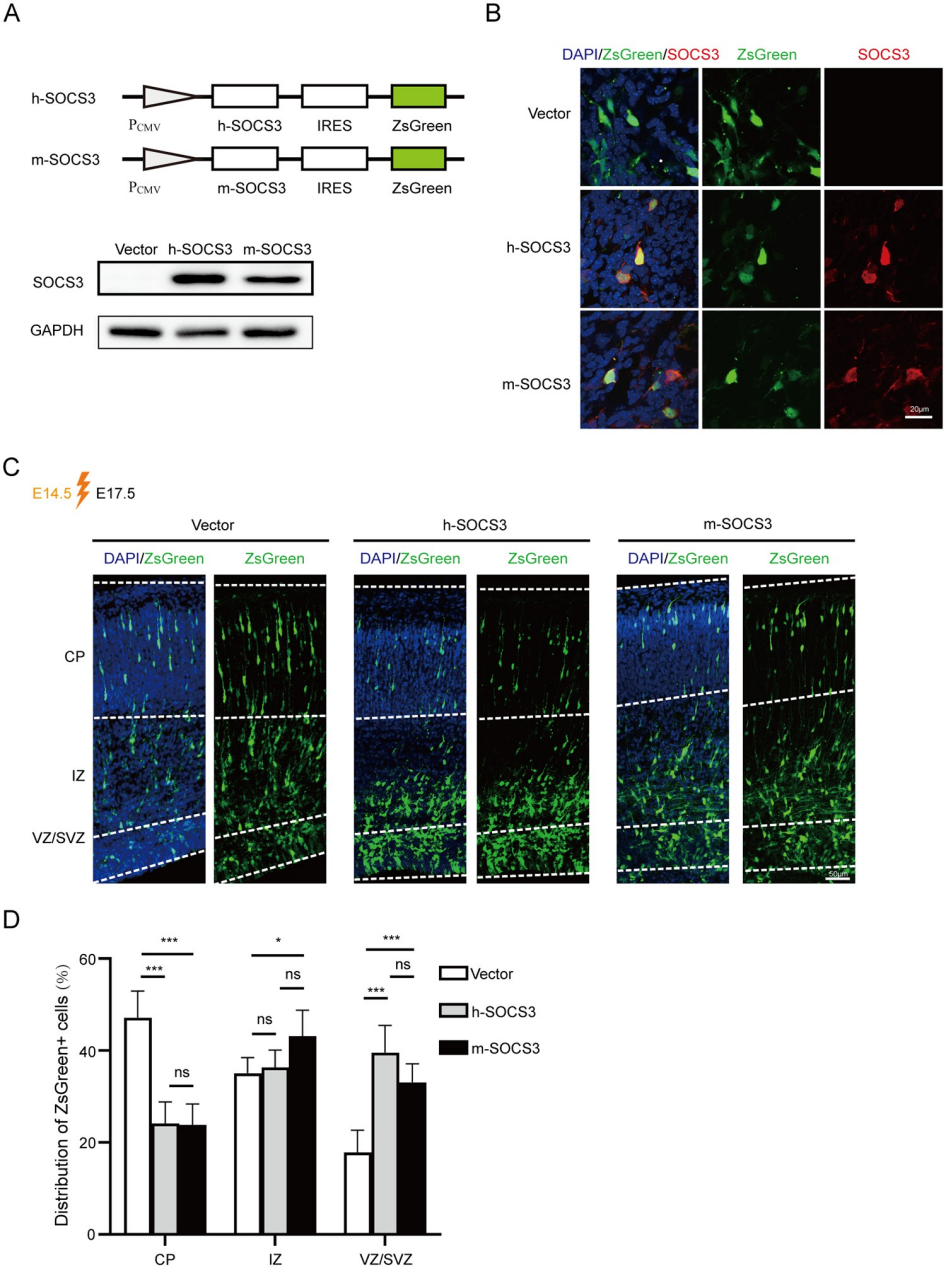
Elevated SOCS3 expression disturbs brain cortex development. **(A)** Diagram of human (h-) and mouse (m-) SOCS3-expressing plasmids, and expression in 293T cells. **(B)** SOCS3 expression in the brains at 3 days post electroporation. h-SOCS3, m-SOCS3, or vector was introduced into the embryonic brain by *in utero* electroporation at E14.5, and brain samples were collected at E17.5. (E14.5 to E17.5, 3 days). Brain tissues were stained with antibodies against SOCS3 (red), and nuclei were counterstained with DAPI (blue). Scale bar, 20μm. **(C)** Representative images of ZsGreen^+^ cells in the brain slides. h-SOCS3, m-SOCS3, or vector was introduced into the embryonic brain by *in utero* electroporation at E14.5, and brains were collected at E17.5. Nuclei were counterstained with DAPI (blue). Scale bar, 50μm. **(D)** Distributions of ZsGreen^+^ cells in the fetal brain cortex. The data were collected from at least five fetuses in each group and analyzed by ANOVA. The results are presented as average ± SD. * p<0.05, *** p< 0.001, ns, no significant difference.

During neurogenesis, NPCs reside and proliferate in the VZ/SVZ, undergo asymmetric cell division and migrate outwards through the intermediate zone (IZ) to the cortical plate (CP), and finally differentiate into mature neurons [34]. We next examined whether elevated SOCS3 expression has a deleterious effect on this process *in vivo*. The SOCS3-expressing plasmid was introduced into the lateral cerebral ventricle of mouse embryos by *in utero* electroporation at E14.5, and the brains were analyzed at E17.5. Human SOCS3 (h-SOCS3) and mouse SOCS3 (m-SOCS3) nucleotide sequences share 91.4% identity, and each was cloned into the vector that co-expresses SOCS3 and a ZsGreen reporter under the same CMV promoter (Fig 8A). Since co-expression of SOCS3 and ZsGreen in cells of the brains was confirmed three days after *in utero* electroporation (Fig 8B), ZsGreen was used as a reporter to track these SOCS3-expressing cells *in vivo*. The cells transfected with the vector control that only expresses ZsGreen exhibited a pattern of gradually increasing proportions of ZsGreen ^+^ cells from the VZ to CP, indicating these cells represent NPCs that migrate out of the VZ. In contrast, ectopic expression of SOCS3 reversed this pattern. Compared with the control, the retention ratio of cells transfected with h-SOCS3 or m-SOCS3 in the VZ/ SVZ was greatly increased, whereas the proportion of cells reaching the CP was reduced markedly (Fig 8C–8D). Notably, we did not observe a significant difference between h-SOCS3 and m-SOCS3 groups, highlighting a conserved role of SOCS3 in neurogenesis between humans and mice. These data suggest that the elevated SOCS3 expression may disturb NPCs maintenance, neuronal migration, and maturation in the fetal brain.

## Discussion

The molecular mechanisms of HCMV-induced abnormal brain development remain poorly characterized. Recently, we developed a new model of congenital MCMV infection that mimics congenital HCMV infection and allows long-term follow-up studies of neurodevelopmental disorders [35]. In this model, congenital CMV infection resulted in cortical atrophy accompanied by impaired NPCs proliferation and migration in the developing brain. However, the molecular mechanisms underlying CMV-induced NPC malfunction needs to be further specified. With the primary human NPC model of HCMV infection, we and others revealed that HCMV infection alters cell fate decisions by intervening with the Notch and STAT3 pathways [4,36]. In the present study, we show that SOCS3, a negative feedback regulator of the STAT3 pathway, is upregulated by pUL97 in NPCs. pUL97 enhances *SOCS3* transcription via a host transcription factor RFX7, probably by phosphorylating RFX7 through its kinase activity. Importantly, elevated SOCS3 expression noticeably hinders NPC migration from the VZ/SVZ to the CP *in vivo*, likely contributing to the observed NPC migration defects in the congenital CMV-infected fetal brain.

Many viruses can stimulate SOCS3 expression to suppress type I interferon responses in a cell type-dependent manner [37]. Different from the typical SOCS3 expression kinetics induced by those viruses, we reveal a biphasic regulation of SOCS3 expression by HCMV infection in NPCs. The first phase resembles the monophasic kinetics of SOCS3 expression stimulated by several viruses, but the stimulated SOCS3 expression was only 3-fold, much weaker than the typical >10-fold by other viruses. These viruses upregulate SOCS3 through distinct mechanisms. For instance, influenza A viruses induce SOCS-3 expression early in the viral replication cycle through nuclear factor kappa B in a type I interferon-independent manner [38]. On the other hand, SOCS-inducing cytokines are involved in the early transient SOCS3 induction by MCMV in mouse IC-21 cells, and UV inactivation renders MCMV incapable of triggering the expression of these cytokines and SOCS3 [39]. Similar to this finding, we did not observe SOCS3 response by UV-inactivated HCMV in NPCs. However, we did not pursue further characterization of the early-phase SOCS3 induction and just focused on the long-sustained second-phase SOCS3 expression with effects on NPC cell fate decisions.

The second phase is characterized by a gradual elevation of SOCS3 level. Since SOCS3 has a short half-life [19,40,41], a constant factor may exist to maintain SOCS3 transcription. In our screening for viral regulators of SOCS3 expression, we not only identified pUL97 as an inducer but also unexpectedly found that IE1 could decrease SOCS3 levels in NPCs. One likely explanation for IE1-mediated SOCS3 downregulation is that IE1 can disrupt the STAT3 pathway in NPCs [4], consequently blocking the downstream activation of *SOCS3* transcription. Thus, SOCS3 expression is determined by the delicate balance of the positive versus negative regulation of pUL97 and IE1, which may explain the weak SOCS3 stimulation by HCMV in NPCs.

As an early-expressed viral protein, pUL97 plays a crucial role in viral replication, and UL97 deletion mutants exhibit severe replication deficits [42]. The viral DNA polymerase processivity factor pUL44 [43] and the major tegument protein pp65 [44] are recognized by pUL97 kinase, indicating the participation of pUL97 in viral DNA synthesis and virion assembly processes, respectively. In addition, pUL97 promotes IE gene expression by disrupting the repressor histone deacetylase 1 with the kinase activity [45]. pUL97 is also a functional ortholog of human cyclin-dependent kinase (CDK)[46]. This peculiar feature of pUL97 enable it to recognize a few CDK substrates (retinoblastoma protein, eukaryotic elongation factor 1delta, lamin A/C, etc.) to alter cell cycle progression and activate protein synthesis, facilitating HCMV replication [47].

Regarding the regulatory mechanism of pUL97, we show that pUL97 enhances SOCS3 transcription through the -1250 to -1000bp region of the *SOCS3* promoter. This region is located far away from the STAT3 binding site (-102 to -45bp) used by the activated STAT3 to drive SOCS3 expression [48,49]. Herpes simplex virus type 1 pUL13, the pUL97 homolog, can also activate SOCS3 expression through the Sp1 region of the *SOCS3* promoter [50]. However, this region is dispensable in our promoter assay, indicating these two proteins act via similar but distinct mechanisms, probably via recruiting different transcription factors to direct the expression. We further demonstrate that the pUL97 kinase activity is required for SOCS3 upregulation. We clearly show that the catalytically inactive pUL97 (K355M) lost the ability to activate the *SOCS3* promoter and induce SOCS3 expression in NPCs. We constructed HCMV-ΔUL97 and HCMV-K355M mutant viruses, but they exhibited serious replication defects and were unsuitable for validation studies. Instead, pUL97 inhibitors MBV and NGIC-I [27,28] were used, and both suppressed SOCS3 upregulation in HCMV-infected NPCs.

Another novel finding in this study is that pUL97 recruits RFX7 for the SOCS3 regulation. RFX7 belongs to the RFX transcription factor family defined by a highly conserved winged-helix type DNA binding domain [51]. RFX7 is prominently expressed in the brain and immune system, regulating genes involved in diverse biological processes such as neural tube closure [52] and natural killer cell homeostasis [53]. A previous study of chromatin state mapping in NPCs showed that RFX family members are highly enriched at neural precursor enhancers, contributing to the global enhancer patterning during neural development [54]. Here we show evidence of RFX7’s participation in pUL97-mediated SOCS3 upregulation in NPCs. First, the co-IP and colocalization assays support direct physical interaction between pUL97 and RFX7. Second, both RFX7 and pUL97 were required to drive the WT and Mut9 *SOCS3* promoter activities. Third, ChIP-qPCR analysis demonstrated that direct binding of RFX7 to the *SOCS3* promoter only occurred in the infected condition and could be reduced by pUL97 inhibitors. Fourth, RFX7 phosphorylation levels were increased by ectopic UL97 expression and HCMV infection in NPCs. Lastly, RFX7 depletion prevented SOCS3 upregulation in HCMV-infected NPCs. One limitation of this study is that we have not been able to purify RFX7 to perform the *in vitro* kinase assay and identify the phosphorylation site due to its large molecular weight and extensive posttranslational modifications [53]. Therefore, whether pUL97 phosphorylates RFX7 directly or through other host factors remains unknown.

SOCS3 has been extensively investigated in immune cells for its role in regulating inflammation [19]. In this study, we provide evidence that elevated SOCS3 expression alters NPC cell fate. The expression patterns of multiple stem cell markers in SOCS3-expressing NPCs are similar to the phenotype in HCMV-infected NPCs, indicating that SOCS3 contributes to cell fate alteration induced by viral infection [4,33]. The decrease of SOX2 and GFAP is likely a consequence of the inhibition of the STAT3 pathway by SOCS3 expression [20,55]. In addition, SOCS3 has been reported to modulate the Notch signaling through Notch1 and Hes5, which may explain the downregulation of other markers [4,20]. Increased SOCS3 level also negatively affects NPC proliferation, neurosphere formation and growth, and migration. Similar to our findings in NPCs, high expression of SOCS3 inhibits migration in hepatocellular carcinoma and lung cancer cells [56–58]. Consistent with the observations in the SOCS3-overexpressing NPCs, ectopic expressing UL97 in NPCs markedly reduced the expression of several NPC markers (S1A Fig) and negatively impacted NPC proliferation (S1B Fig), neurosphere formation (S1C Fig) and migration (S1D Fig). These matched alterations provide a link between pUL97 and SOCS3 in altering NPC properties.

We further explored whether elevated SOCS3 expression interferes with NPC migration *in vivo*. SOCS3-expressing vector was introduced into the lateral ventricle by *in utero* electroporation at E14.5. At this point, the fetal brain is undergoing neurogenesis, and thus the target cells are mainly NPCs in the VZ/SVZ. Later, these NPCs will migrate toward the CP and differentiate to mature neurons [34]. The fetal brain images showed impaired migration of the SOCS3-expressing NPCs, with far fewer cells reaching the CP than NPCs carrying the control vector. This finding suggests the altered SOCS3 expression by pUL97 contributes to the observed brain maldevelopment in the congenital CMV infection model.

**Fig 9:**
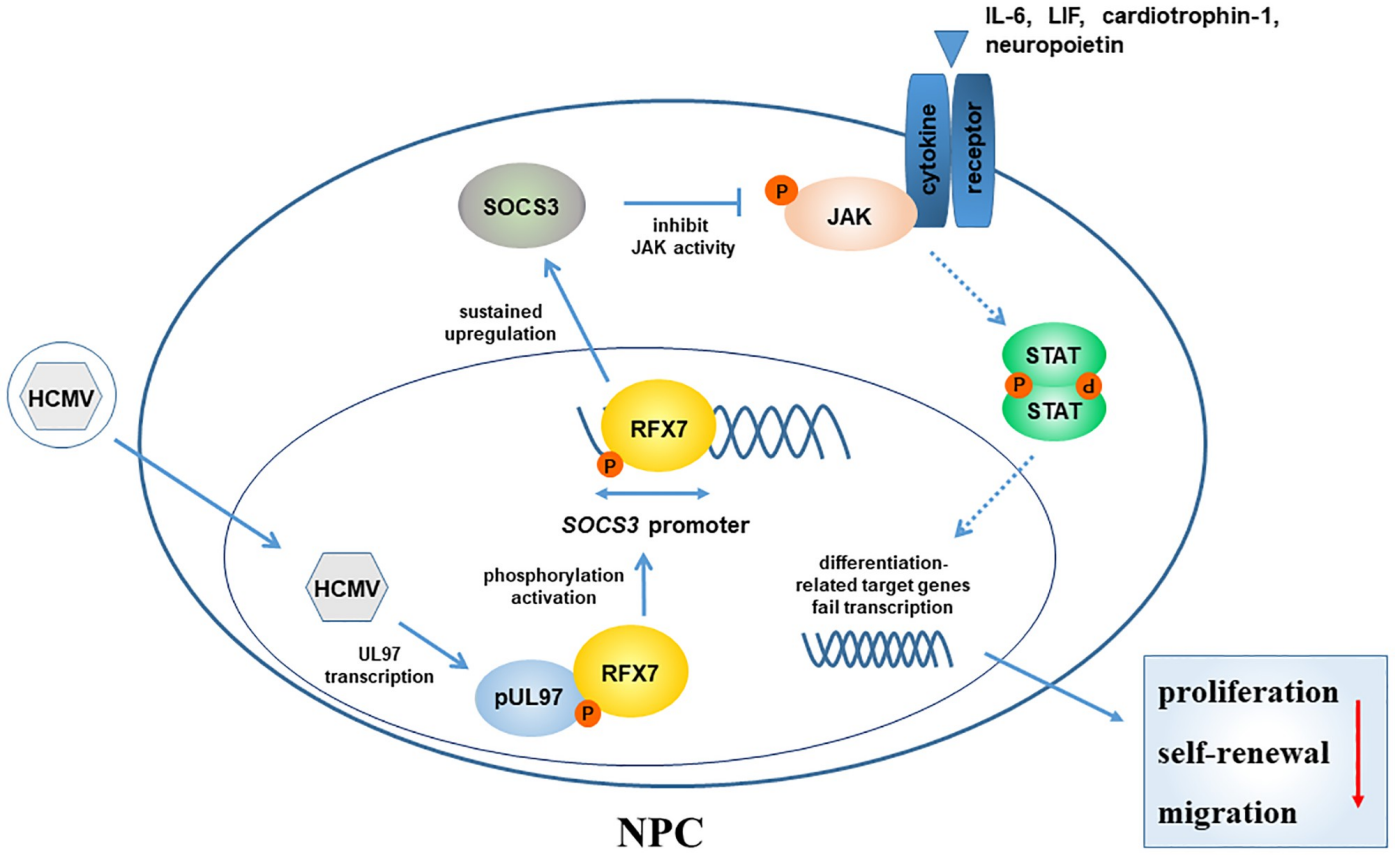
A working model for HCMV-induced SOCS3 upregulation in NPCs. HCMV-encoded pUL97 activates RFX7 by phosphorylation in infected NPCs. The activated RFX7 upregulates SOCS3 transcription through its promoter. Elevated SOCS3 expression suppresses the IL6-JAK-STAT pathway. This blockage disrupts NPC’s response to extracellular neurodevelopment cues and consequently results in impaired self-renewal, proliferation, and migration of NPCs.

In summary, this study provides a mechanism for pUL97-mediated SOCS3 upregulation induced by HCMV infection in NPCs and reveals the negative impacts of sustained SOCS3 expression on neurogenesis (Fig 9). These findings may help to elucidate how congenital CMV infection causes fetal brain maldevelopment.

## Materials and methods

### Ethics statement

Human neural progenitor cells (NPCs) were isolated from postmortem fetal embryos at different gestational age, and maintained in our laboratory. The cell isolation procedures and research plans are approved by the Institutional Review Board (IRB, WIVH10201202) according to the Guidelines for Biomedical Research Involving Human Subjects at Wuhan Institute of Virology, Chinese Academy of Sciences. The need for written or oral consent was waived by IRB. All mouse experiments were approved by the Wuhan Institute of Virology Institutional China of Ethics Committee on Animal Experiments (WIVA10201504) and were performed following the Guide for the Care and Use of Laboratory Animals as published by the US National Institutes of Health (National Academies Press, 2011).

### Cells and cell culture

NPCs were isolated and maintained in the lab as described [5]. Briefly, the growth medium (GM) for culturing NPCs was Dulbecco’s Modified Eagle Medium (DMEM-F12, Thermo Fisher Scientific) supplemented with GlutaMAX (2 mM, Thermo Fisher Scientific), penicillin and streptomycin (100 U/ml and 100 μg/ml, Thermo Fisher Scientific), gentamycin (50g/ml, Sigma), amphotericin B (1.5g/ml, Thermo Fisher Scientific), 10% BIT 9500 (Stem Cell Technologies), epidermal growth factor (EGF, 20 ng/ml, Prospec), and basic fibroblast growth factor (FGF, 20 ng/ml, Prospec). For maintenance, the NPCs culture medium was replaced with half of fresh GM every two days. The replaced medium was collected after the removal of cell debris by centrifugation and used as a conditioned medium (CM).

Human embryonic kidney (HEK) 293T cells (CRL-11268) and NIH3T3 were cultured in Dulbecco’s modified Eagle medium (DMEM, Life Technologies) with 10% fetal bovine serum (FBS, Gibco BRL), 100 U/ml penicillin and 100 μg/ml streptomycin. Human foreskin fibroblasts (HFFs) were cultured in minimal essential medium (MEM, Life Technologies) supplemented with 10% FBS and penicillin-streptomycin as described above. All cell cultures were performed at 37°C in a humidified atmosphere containing 5% CO_2_.

### Plasmid constructs and lentiviruses

The coding sequences of human SOCS3 (GenBank Gene ID: 9021), Mus musculus SOCS3 (GenBank Gene ID: 12702), and human RFX7 (GenBank Gene ID: 64864) were cloned into pHAGE-CMV-MCS-EF1-copZsGreen (pHAGE-ZsGreen; Addgene) to generate pHAGE-h-SOCS3, pHAGE-m-SOCS3, pHAGE-RFX7 and pHAGE-RFX7-HA. HCMV IE1 (UL123), IE2 (UL122), pp65 (UL83), UL26, UL97, and K355M were cloned into the vector to generate pHAGE-IE1, pHAGE-IE2, pHAGE-pp65, pHAGE-UL26, pHAGE-UL97, pHAGE-K355M, pHAGE-UL97-FLAG and pHAGE-K355M-FLAG. shRNAs sh-Scram (5′-TTCTCCGAACGTGTCACGT-3′), sh1-UL97 (5′-TTGGCCGACGCTATCAAATTT-3′), sh2-UL97 (5′-GTGTATGCCACTTTGATATTA-3′), sh3-UL97 (5′-ACGAGCGACGGGCTGTATTTA-3′), sh1-RFX7 (5′-TGAAGGT GTCATTGAAATAAA-3′), sh2-RFX7 (5′-CATCATCATCTCCAGATATAA-3′), and sh3-RFX7 (5′-GATACAGGAATCTTCTTTAAA-3′) were cloned into lentiviral vector pCDSHR (constructed based on pCDH-CMV-MCS-EF1α-puro in our laboratory).

pGL3-Basic (Addgene) was used to construct various *SOCS3* promoter truncates, including pGL3-*SOCS3*-promoter (WT, Mut 1–9).

Lentiviruses expressing SOCS3 or viral genes were produced in HEK293T cells [59]. In brief, HEK293T cells were seeded onto 10-cm dishes and cotransfected with 10 μg lentiviral vector DNAs, 10 μg helper plasmids pMD2.G, and 10 μg psPAX2, using the calcium phosphate transfection method. At 24 h post-transfection, the culture medium was replaced with fresh medium without antibiotics. At 48 h and 72h, lentiviruses were harvested with filtration, titrated, and stored at −80°C.

Lentiviruses expressing shRNAs were produced in HEK293T cells as described previously [4,5]. HEK293T cells were cotransfected with 15 μg lentiviral vector DNAs, 12 μg helper plasmids pML-Δ8.9, and 8 μg pVSV-G, and the virus was harvested as described above.

### Viruses and infection

The HCMV Towne strain (ATCC VR-977) and MCMV K181-eGFP strain were used in this study [35,60]. HCMV and MCMV were propagated and titrated as described previously. In brief, HCMV and MCMV were respectively propagated and harvested in HFF and NIH3T3 cells. The virus particles in the cell supernatants were concentrated by ultracentrifugation to remove cell debris and potential undesired component, and resuspended in DMEM-F12 without FBS as described previously [4,5]. UV-inactivated HCMV was prepared by irradiation of 6000 J/m^2^ three times in CL-1000 UV cross-linker (UVP) with added sodium pyruvate (5mM) to prevent free radical damage.

NPCs were digested by Accutase (Millipore), reseeded in poly-D-lysine -coated dishes or coverslips, and allowed to attach overnight. NPCs were incubated with HCMV for 3 h to allow virus adsorption, and then the inoculum was removed and replaced with a fresh medium. The Infected cells were collected and analyzed at the indicated time post-infection. To explore the effect of virus replication on SOCS3 expression, NPCs were pretreated with GCV (117μg/ml) and PAA (100μg/ml) for 1 h before infection, and the medium was replaced every 24 h to maintain drug concentration. To study the effect of pUL97 kinase activity on SOCS3 expression during infection, NPCs were treated with kinase inhibitor MBV (40μM) or NGIC-I (1μM), similar to the GCV and PAA treatment. CHX (10μg/ml) (Sigma) was added for 1 h prior to infection to block *de novo* protein synthesis during infection.

### Target gene expression and silencing

Ectopic gene expression in NPCs was achieved by nucleofection or lentiviral transduction, as described previously [5]. Nucleofection (Lonza) was used according to the manufacturer’s instructions. Briefly, 5×10^6^ NPCs were mixed with 100 μl Mesenchymal Stem Cell (MSC) Nucleofector Solution (82 μl Nucleofector Solution with 18 μl Supplement 1) containing plasmid DNA. The mixture was transferred to certified cuvettes, and the transfection was carried out in a Nucleofector II using Nucleofector Program A-033. After nucleofection, NPCs were gently resuspended with 1ml GM/CM (1:1) and reseeded to poly-D-lysine-coated dishes. The medium was changed 12h later, and the NPCs were collected 48 h post-transfection for further experiments. NPCs were transduced with lentiviruses at an MOI of 1 and cultured for 48 h to allow target gene or shRNA expression.

### Western blotting

Cells or brain tissues were collected and lysed with lysis buffer (Beyotime) containing protease inhibitor (Roche). The protein concentration was measured using the Bradford assay (Bio-Rad). The lysates samples with equal amounts of total protein were separated by SDS-PAGE electrophoresis (PAGE) and then transferred to polyvinylidene fluoride membranes (PVDF; Millipore). After blocking with 5% skim milk-TBST for 1 h, the indicated targets were probed with primary antibodies and appropriate peroxidase-conjugated secondary antibodies. The antibodies used in the experiments include anti-IE1 [61] and anti-pUL97 (gift from William J. Britt, University of Alabama, USA)[62], anti-IE1/2 (p1215, Virusys), anti-pp65 (p1205, Virusys), anti-UL44 (p1202-1, Virusys), anti-pp28 (CA004-1,Virusys), anti-mIE1 [63], anti-GAPDH (10494-1-AP, Protientech), anti-FLAG (20543-1-AP, Protientech), anti-RFX7 (NBP1-71819, Novus), anti-SOCS3 (ab16030, Abcam), anti-GFAP (16825-1-AP, Proteintech), anti-Nestin (19483-1-AP, Proteintech), anti-DCX (13925-1-AP, Proteintech), anti-SOX2 (ab97959, Abcam), Phospho-(Ser/Thr) Substrate Antibody (9611S, Cell Signaling Technology), anti-STAT3 (10253-2-AP, Proteintech) and anti-Phospho-STAT3 (Tyr705) (9145, Cell Signaling Technology). The protein bands were detected using a Chemiluminescence machine and analyzed by a densitometry program (Image J).

### Immunofluorescent assay

NPCs were seeded on poly-D-lysine-coated coverslips in 12-well plates and infected with HCMV after attachment. The coverslips were harvested and fixed with 4% paraformaldehyde at the indicated times post-infection. PBS-perfused Brains were fixed in 4% paraformaldehyde and prepared for IFA. anti-SOCS3 (IgG2b; ab14939, Abcam), anti-SOCS3 (Rabbit; ab16030, Abcam), anti-HA (Rabbit; Covance) and anti-IE1/2 (IgG1; Virusys) were detected by primary antibodies and appropriate secondary antibodies [35,64]. The secondary antibodies included TRITC-mouse IgG2b (1090–03, Southern Biotech), Alexa Fluor 488-mouse IgG1 (A-21121, Invitrogen), Alexa Fluor 647-rabbit (A-21247, Invitrogen), and Nuclei were counterstained with DAPI (4′,6-diamidino-2- phenylindole) (D9542, Merck).

### Immunoprecipitation assay

The cells were collected by centrifugation and re-suspended in Cell Lysis Buffer (Beyotime). After removing debris, cell lysates containing 1 mg total protein were incubated with 1μg antibody overnight at 4°C. Protein A+G agarose beads (30μl, Beyotime) were added and rotated for 3 hours at 4°C. The beads were collected after centrifugation at 1000g for 1 min and washed 5 times by Cell Lysis Buffer. Then the IP complexes were denatured with the loading buffer at 100°C for 5mins, loaded to SDS-PAGE, and immunoblotted (IB) for the indicated targets. The antibodies in the IP assay included anti-FLAG-Mouse (F1804, Sigma), and anti-RFX7 (NBP1-71819, Novus).

### Quantitative reverse transcriptase PCR

Total RNA was extracted from transfected, infected cells and brain tissues using the RNAiso Plus reagent (Takara). PrimeScript RT Reagent Kit with gDNA Eraser was used for genome digestion and reverse transcription following the manufacturer’s instructions (TaKaRa). Target genes were determined by the CFX-96 Connect system (Bio-Rad) with SYBR Green (Bio-Rad). The specific primers are listed in S2 Table. Three independent experiments were performed, and target genes were normalized to GAPDH with the 2^ΔΔCT^ method.

#### Chromatin Immunoprecipitation (ChIP)-quantitative PCR (qPCR) assay

RFX7 ChIP-qPCR assays were performed as previously described [65]. Briefly, NPCs were harvested and fixed with 1% formaldehyde for 20 min to cross-link DNA and proteins. Fixation was quenched by adding glycine. Chromatin was sheared using Qsonica (Q125) (20 cycles of sonication: 10 s ON; 30 s OFF; and AMPL, 80%). Chromatin (2 ug) was then incubated with RFX7 antibody (NBP1-71819, Novus) and immunoglobulin G (IgG) antibody (negative control; 2729, CST), respectively, and immunoprecipitated by protein G magnetic beads (20 ul). After extensive washing [RIPA-LS (low salt), RIPA-HS (high salt), RIPA-LiCl, and 10 mM tris (pH 8)], protein-DNA complexes were eluted, and cross-linking was reversed by adding 6 ul of 5 M NaCl and 2 ul proteinase K and incubated overnight at 65°C. DNA was purified and subjected to qPCR for DNA enrichment detection. The primers are listed in S2 Table. The results showed as follows: 2% × 2^(CTinput−CTRFX7)^/[2% × 2^(CTinput−CTIgG)^].

### Mice, infection, and *in utero* electroporation

The 8 weeks old ICR mice were used in this study. Pregnant females were obtained by mating for 12 hours and identified by vaginal plugs, and the fetuses were selected for MCMV infection or *in utero* electroporation. For MCMV infection, 2 μl of MCMV was injected into the lateral ventricle of E13.5 fetuses, and the fetuses injected with DMEM medium were considered the mock control [35]. The fetuses were harvested through cesarean section at E18.5 or P0 (natural birth newborns). For *in utero* electroporation, 0.05% fast green (Sigma) was added to plasmid DNA dissolved in 1×PBS (2 μg/μl), and 1–2 μl of plasmid solution was injected into the lateral ventricle of E14.5 fetus brain with capillary tubing. After the DNA injection, 5 square pulses (33V 50msec-on 1sec-off) were delivered to the brains with platinum electrodes (5mm diameter) [66,67] using the BTX Electro ECM830 square wave electroporation system.

### Luciferase reporter assay

The 293T cells were seeded in 24-well plates and co-transfected with pHAGE- UL97/K355M/RFX7/vector and different SOCS3 promoter truncate-carrying luciferase reporter plasmids or shRNAs, using LipoFectMax transfection reagent (ABP Biosciences). At 36h post-transfection, the cells were harvested, and luciferase activity was detected by Luciferase Assay System (Promega).

### Proliferation and cell viability assays

NPCs were transduced with SOCS3-expressing or control lentivirus and cultured for 48 h. Then NPCs were reseeded at 5×10^4^ cells/well in poly-D-lysine-coated 96-well plates, and cell viability was analyzed by the WST-1 assay on days 1, 3, and 5. The WST-1 reagent (10 μl, Beyotime) was added to each well (100 μl) and incubated at 37°C for 2 h. The absorbance at 450 nm was determined with 690 nm as the reference using an Epoch microplate spectrophotometer (BioTek Instruments, USA).

### The scratch assay

NPCs were transduced with SOCS3-express or the control lentivirus, cultured for 48 h, and reseeded at 1×10^6^ cells/well in poly-D-lysine-coated 24-well plates, using the 20 μl plastic pipette tips to create the scratch area, and the floating and dead cells were cleared by GM wash. During the scratch assay, NPCs were cultured with GM, and the fields were photographed using Nikon Eclipse TS100 with a Nikon CoolPix P6000 camera at the times indicated. The area of the scratch was analyzed by a densitometry program (Image J).

### Neurosphere formation assay

After transduction with SOCS3-expressing or the control lentivirus, NPCs were cultured for 48 h and then digested and reseeded at a density of 1×10^6^ cells/well in uncoated 6-well plates. Images from three random fields for each sample were taken after 48 h culture. The neurospheres were categorized into three size groups (small, < 50 μm; medium, 50–100 μm; and large, > 100 μm) by Image J.

## Supporting information

S1 Fig Ectopic expression of UL97 alters NPC properties. **(A)** Effect of pUL97 on the expression of key NPC markers. NPCs nucleofected with UL97 or vector were cultured for 48 h and then harvested for the indicated proteins examination by western blotting. GAPDH served as a loading control. **(B)** Effect of pUL97 on cell viability. NPCs were transduced lentivirus expressing UL97 or the control for 48h and then reseeded in poly-D-lysine-coated 96-well plates. NPC proliferation was assessed at 1 and 3 days by WST-1 assay. **(C)** Effect of pUL97 on neurosphere formation and growth. At 48h post-transduction, the NPCs were reseeded in uncoated 6-well plates, and images were obtained after culturing for 48 h. All neurospheres were counted and categorized into three size groups (small, < 50μm; medium, 50–100μm; and large, > 100μm) from 3 random fields. Scale bars, 100μm. **(D)** Effect of pUL97 on NPC migration. At 48h post-transduction, NPCs were reseeded in poly-D-lysine-coated 24-well plates for further culture. When cells reached confluency, a cell-free zone was created with pipette tips, and the floating and dead cells were cleared by GM wash. The same fields were imaged every 24 h and analyzed by the densitometry program (Image J). The scratch areas at the indicated times were normalized to the measured area at 0 h. Scale bar, 100μm. Data in (**A**–**B**) are from three independent experiments, and data in (**C**–**D**) are from three random fields or sights. All data are presented as average ± SD. Significance was tested with the student’s t-test; * p<0.05, *** p<0.001.
(TIF)

S1 Table pUL97-interacting proteins identified by LC-MS/MS. (XLSX)

S2 Table qRT-PCR primers used in this study. (DOCX)
