# Supplementary figures and images for: Human cytomegalovirus pUL97 upregulates SOCS3 expression via transcription factor RFX7 in neural progenitor cells
Source: PLoS Pathog. 2023 Feb 8;19(2):e1011166. doi: 10.1371/journal.ppat.1011166 (PMC9942973; doi:10.1371/journal.ppat.1011166)

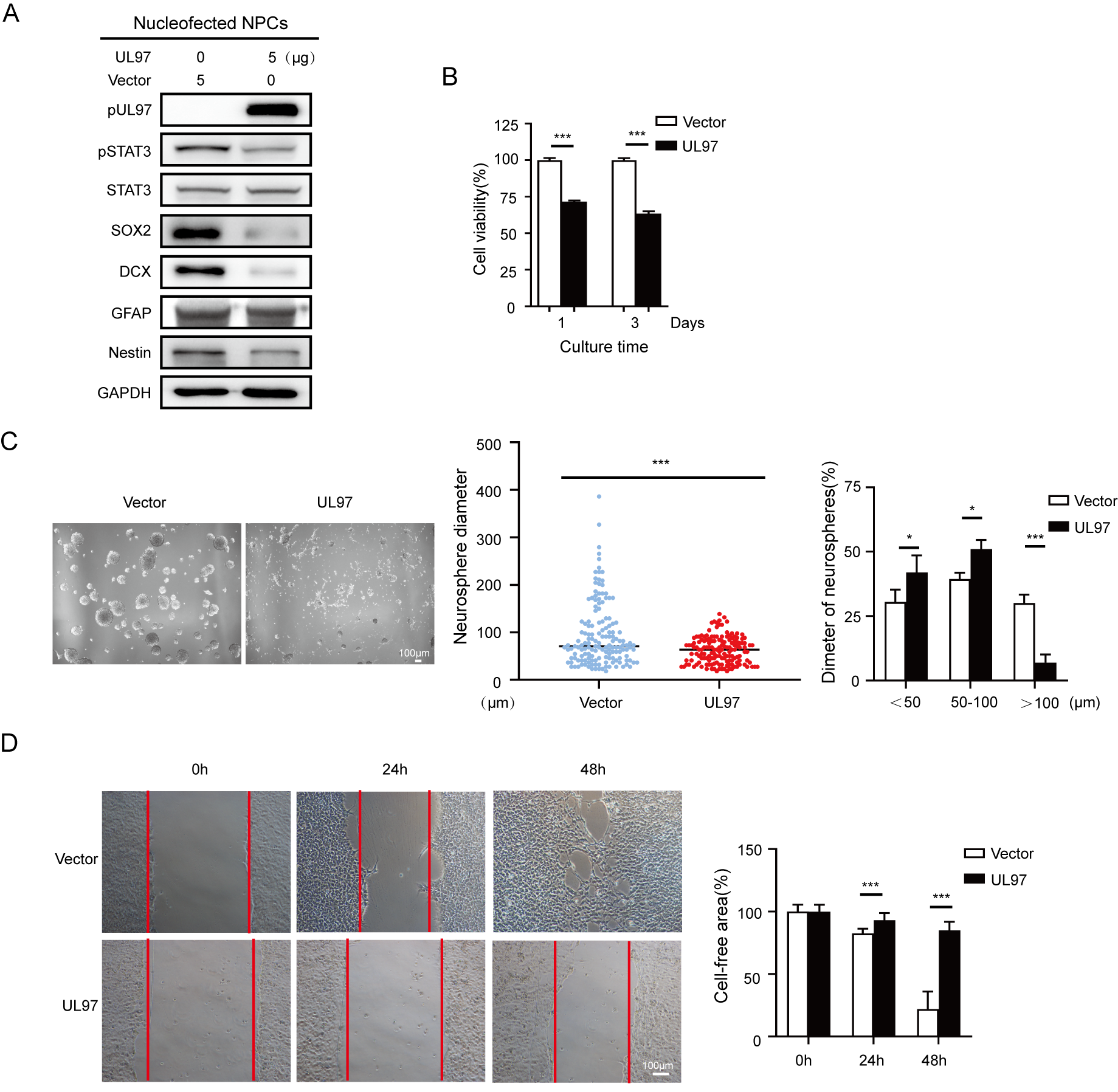

Supplement: S1 Fig — (A) Effect of pUL97 on the expression of key NPC markers. NPCs nucleofected with UL97 or vector were cultured for 48 h and then harvested for the indicated proteins examination by western blotting. GAPDH served as a loading control. (B) Effect of pUL97 on cell viability. NPCs were transduced lentivirus expressing UL97 or the control for 48h and then reseeded in poly-D-lysine-coated 96-well plates. NPC proliferation was assessed at 1 and 3 days by WST-1 assay. (C) Effect of pUL97 on neurosphere formation and growth. At 48h post-transduction, the NPCs were reseeded in uncoated 6-well plates, and images were obtained after culturing for 48 h. All neurospheres were counted and categorized into three size groups (small, < 50μm; medium, 50–100μm; and large, > 100μm) from 3 random fields. Scale bars, 100μm. (D) Effect of pUL97 on NPC migration. At 48h post-transduction, NPCs were reseeded in poly-D-lysine-coated 24-well plates for further culture. When cells reached confluency, a cell-free zone was created with pipette tips, and the floating and dead cells were cleared by GM wash. The same fields were imaged every 24 h and analyzed by the densitometry program (Image J). The scratch areas at the indicated times were normalized to the measured area at 0 h. Scale bar, 100μm. Data in (A–B) are from three independent experiments, and data in (C–D) are from three random fields or sights. All data are presented as average ± SD. Significance was tested with the student’s t-test; * p<0.05, *** p<0.001. (TIF) [file ppat.1011166.s001.tif]
