# Supplementary material for: Human cytomegalovirus pUL97 upregulates SOCS3 expression via transcription factor RFX7 in neural progenitor cells
Source: PLoS Pathog. 2023 Feb 8;19(2):e1011166. doi: 10.1371/journal.ppat.1011166 (PMC9942973; doi:10.1371/journal.ppat.1011166)
Supplement: S2 Table — (DOCX) [file ppat.1011166.s003.docx]

Supplementary Table 2. qRT-PCR primers used in this study

| Plasmids | Primers |
| --- | --- |
| SOCS3 | **F** 5’-GAGCCCCCTCCTTCCCCTCGC-3’  **R** 5’-GGTCCAGGAACTCCCGAATG-3’ |
| RFX7 | **F** 5’-TGATGATGAACTTACACAA-3’ |
|  | **R** 5’-TTACTGACTGACTCTGAA-3’ |
| UL97 | **F** 5’-GGCTCCTTCGGCGAGGTCTGG-3’  **R** 5’-GCGACGGCGGCTGCTGTTG-3’ |
| human-GAPDH | **F** 5’-GATGACATCAAGAAGGTGGTG-3’  **R** 5’-GTCTACATGGCAACTGTGAGG-3’ |
| mouse-GAPDH  CHIP-SOCS3 *promoter* 1  CHIP-SOCS3 *promoter* 2  CHIP-SOCS3 *promoter* 3 | **F** 5’-TGGCCTTCCGTGTTCCTAC-3’  **R** 5’-GAGTTGCTGTTGAAGTCGCA-3’  **F** 5’-actaaagtgcagtggcac-3’  **R** 5’-ttcaagaccagcctgg-3’  **F** 5’-tgtccatgctcaagtgat-3’  **R** 5’-ttgccactgtatcctc-3’  **F** 5’-aggcagtgcgcacctat-3’  **R** 5’-tttcacctctcgggat-3’ |
| CHIP-SOCS3 *promoter* 4 | **F** 5’-atctaaatgccactga-3’  **R** 5’-ttggagacccggggcc-3’ |
| CHIP-SOCS3 *promoter* 5 | **F** 5’-CCAAGCCCAAACTATCTA-3’  **R** 5’-GGAGAGGAAGTGATTGAG-3’ |
| CHIP-SOCS3 *promoter* 6 | **F** 5’-tcaatcacttcctctc-3’  **R** 5’-agatggcccacaccccct-3’ |
| CHIP-SOCS3 *promoter* 7 | **F** 5’-atctggcagtagcctgaa-3’  **R** 5’-attcacaccccaggct-3’ |
| CHIP-SOCS3 *promoter* 8 | **F** 5’-atgcccccttctcggc-3’  **R** 5’-tggagagctcgaggtgga-3’ |
| CHIP-SOCS3 *promoter* 9 | **F** 5’-gcccggccgagagcac-3’  **R** 5’-attccagaggggagac-3’ |
| CHIP-SOCS3 *promoter* 10 | **F** 5’-ctgcccgcaggtgact-3’  **R** 5’-ttttcccggaccccgc-3’ |
| CHIP-SOCS3 *promoter* 11 | **F** 5’-aaaaggggaaggggaa-3’  **R** 5’-aacttctcattcacac-3’ |
| CHIP-SOCS3 *promoter* 12 | **F** 5’-agaagttgggggcgga-3’  **R** 5’-aaaggctgagcgcgga-3’ |
| CHIP-SOCS3 *promoter* 13 | **F** 5’-tttctctgctgcgagt-3’  **R** 5’-ggtatataggcggccg-3’ |
|  |  |
